# Mobile applications in medical education: A systematic review and meta-analysis

**DOI:** 10.1371/journal.pone.0265927

**Published:** 2022-03-24

**Authors:** Viji Pulikkel Chandran, Athira Balakrishnan, Muhammed Rashid, Girish Pai Kulyadi, Sohil Khan, Elsa Sanatombi Devi, Sreedharan Nair, Girish Thunga

**Affiliations:** 1 Department of Pharmacy Practice, Manipal College of Pharmaceutical Sciences, Manipal Academy of Higher Education, Manipal, Karnataka, India; 2 Department of Pharmaceutics, Manipal College of Pharmaceutical Sciences, Manipal Academy of Higher Education, Manipal, Karnataka, India; 3 School of Pharmacy and Medical Sciences, Quality Use of Medicines Network, Menzies Health Institute, Griffith University, Gold Coast, Queensland, Australia; 4 Department of Medical Surgical Nursing, Manipal College of Nursing, Manipal Academy of Higher Education, Manipal, Karnataka, India; University of Dhaka, BANGLADESH

## Abstract

**Objective:**

This review evaluates the effectiveness of smartphone applications in improving academic performance and clinical practice among healthcare professionals and students.

**Methods:**

This study followed the Preferred Reporting Items for Systematic Reviews and Meta-Analyses (PRISMA) guidelines. Articles were retrieved from PubMed, Scopus, and Cochrane library through a comprehensive search strategy. Studies that included medical, dental, nursing, allied healthcare professional, undergraduates, postgraduates, and interns from the same disciplines who used mobile applications for their academic learning and/or daily clinical practice were considered.

**Results:**

52 studies with a total of 4057 learner participants were included in this review. 33 studies (15 RCTs, 1 cluster RCT, 7 quasi-experimental studies, 9 interventional cohort studies and 1 cross-sectional study) reported that mobile applications were an effective tool that contributed to a significant improvement in the knowledge level of the participants. The pooled effect of 15 studies with 962 participants showed that the knowledge score improved significantly in the group using mobile applications when compared to the group who did not use mobile applications (SMD = 0.94, 95% CI = 0.57 to1.31, P<0.00001). 19 studies (11 RCTs, 3 quasi-experimental studies and 5 interventional cohort studies) reported that mobile applications were effective in significantly improving skills among the participants.

**Conclusion:**

Mobile applications are effective tools in enhancing knowledge and skills. They can be considered as effective adjunct tools in medical education by considering their low expense, high versatility, reduced dependency on regional or site boundaries, online and offline, simulation, and flexible learning features of mobile apps.

## Introduction

Applications or apps are software programs designed to run on a computer/tablet/mobile phone to accomplish a particular purpose. Mobile applications play an integral role in medical education, as healthcare professionals (HCPs) and students use these emerging technologies during their training and practice. They have become inevitable in clinical educational settings, particularly as they are accessible for learning anywhere. Many applications are designed to support HCPs with significant tasks such as documentation and time management, health record management and access, consulting and networking, information and reference acquisition, clinical care and monitoring, medical education, training, and clinical decision making [1]. Apps are popular as they have a multimedia approach and include images, videos, texts, and podcasts [2].

The medical education framework encompasses a highly standardized curriculum in a range of preclinical and clinical settings. The designs and specifications of this framework are defined by the Medical Education Board of every country. Competent HCPs are not born; they are educated to combine the art of science with recent concepts of illness, diagnosis, treatment, and empathy. Education in medical institutions endured abrupt disturbances in the face of the 2019 coronavirus pandemic (COVID-19) [3]. There is ambiguity surrounding how long the condition will continue to exist [4]. Since the pandemic, educational experts are realizing that the implementation and administration of medical curricular modifications will be versatile, building on the current pedagogical framework [5].

In today’s context, as mobile apps offer new learning opportunities, mobile learning is emerging as the newer form of learning and implemented as a result of this pandemic. Most experts suggest that mobile learning initiatives will significantly enhance the learning processes in healthcare. Several randomized trials of mobile learning approaches that aimed at improving knowledge, skills, attitude, and satisfaction have been released. Nevertheless, these results were not carefully checked, and there was no quantification in the effectiveness of mobile learning interventions [6]. Studies suggest that more than 85% of HCPs and medical students use a smartphone, and 30–50% use medical apps for learning and collecting information [7]. The field of medical applications development is very diverse and many applications are being designed to fulfill the needs of medical students and HCPs. As time progressed, smartphones and mobile apps have started to replace conventional knowledge acquisition settings and provide medical students with unparalleled ease of access to medical information and expertise [8]. Despite this acceptance, there is limited research that substantiates the claim that the use of smartphones is effective in enhancing the academic performance of HCPs and/ or students [9].

Mobile apps have a relatively low expense, high versatility, and reduced dependency on regional or site boundaries which encourage stakeholder investments (countries, networks, and universities) and learner demands. This review aims to evaluate the effectiveness of smartphone-based applications in improving academic performance and clinical practice among healthcare professionals and/or students by assessing the impact of the use of smartphone-based interventions and applications in knowledge acquisition and skill levels.

## Materials & methods

This study followed the Preferred Reporting Items for Systematic Reviews and Meta-Analyses (PRISMA) Guidelines for reporting the findings. The study protocol was registered in PROSPERO: International Prospective Register of Systematic Reviews with the registration number: CRD42019133670 [10] before conducting the study.

### Criteria for inclusion of studies

#### Type of studies

Quantitative studies such as randomized controlled trials (RCTs), cluster RCTs (cRCTs), quasi-experimental studies, interventional cohort studies, and cross-sectional studies that assessed the change in knowledge or skill following the use of any mobile application in the study population were included. We excluded studies that assessed the perception of the use of mobile applications, non-English publications, editorials, reviews, conference proceedings, qualitative studies and those on the design and development process.

#### Type of participants

Medical, dental, nursing, allied HCPs, undergraduates, postgraduates, and internship students from the same disciplines who used mobile applications for academic learning and/or daily clinical practice were included. Participants were not excluded based on their age, gender, experience, and any other socio-demographic characters.

#### Types of intervention

We included studies that assess the effectiveness of online/offline mobile applications in the acquisition of knowledge and skill development. Studies that include mobile applications related to drug information, guidelines, health parameter calculators, diagnosis, disease, medical notes, case studies, and simulation-aided models to improve knowledge and skills in practice as an intervention were also included. Any intervention using stationary technology, such as desktop computers, e-learning courses attended through internet platforms on mobile phones/tablets/computers, and mobile applications on non-medical topics related to knowledge and skill development among HCPs/ students were excluded.

#### Types of outcome measures

We included studies that assessed quantitative outcomes in terms of knowledge or skill level among HCPs/students.

### Search methods for identification of studies

#### Electronic & other resources searches

We retrieved articles from PubMed, Scopus, and Cochrane library databases using a comprehensive search strategy from inception till December 2020. We identified all the possible keywords and medical subject heading (MeSH) terms for the terms “healthcare professionals”, “doctors”, “nurses”, “pharmacists”, “allied healthcare professionals”, “paramedical professionals” and “mobile apps” from the previously published studies and databases. We also used the truncation search method (*) to avoid the possibility of missing studies due to changes in terms in different studies. Further, we searched Google, bibliography of included studies, and relevant reviews published in the same area to look for any other additional studies. The detailed search strategy in different databases is provided in the S1 Appendix.

#### Selection of studies and data extraction

Two review authors independently determined the eligibility of the studies by examining the title and abstract of the retrieved studies based on the pre-defined inclusion and exclusion criteria, followed by full-text screening. Two review authors independently extracted data from the included studies, using a data extraction form developed using Microsoft Excel. The following information was extracted: characteristics of study (authors name, year of publication, study location), characteristics of the population (type of healthcare professionals/students [year of professional education]), study design, number of participants in study groups at baseline and at the time of completion, characteristics of intervention and/ or control (name and content details of the mobile app, nature of control), outcome measures (scale used), result (knowledge/skill score in mean and standard deviation (SD)/ standardized mean difference (SMD) and SD/median/P value) and limitations of the included studies. All the disagreements during the study selection and data extraction were resolved by consensus or discussion with another researcher.

#### Assessment of risk of bias in the included studies

The quality of RCTs was assessed using the Cochrane risk of bias assessment tool [11] and other study designs were (interventional and observational) assessed using the Modified New castle Ottawa scale [12]. Cochrane risk of bias assessment tool assesses the following criteria of studies: (i) random sequence generation, (ii) allocation concealment, (iii) blinding of participants and personnel, (iv) blinding of outcome assessment, (v) incomplete outcome data, (vi) selective reporting and (vii) any other bias not included in other types into high risk, low risk and unclear risk categories. Modified New castle Ottawa scale assesses the following criteria: (i) representativeness of intervention group (1 point) (ii) selection of comparison group (1point) (iii) comparability of the comparison group (2 points) (iv) study retention (1 point) (v) blinding of assessment (1 point), totaling a maximum of 6 points. Two independent reviewers were involved in the quality assessment and any disagreements were resolved by discussion and consensus.

#### Measures of mobile application effectiveness

We separately analyzed the change in knowledge and skill acquisition by using mobile applications among the HCPs and/or students. Our analysis was based on the consideration of continuous outcome variables (change in participants’ knowledge and/or skill score). Studies assessing both skill and knowledge were reported separately in both domains with relevant outcome details.

#### Data synthesis

The characteristics of the included studies and outcome measures collected were presented in a tabular form and a narrative synthesis was performed. The meta-analysis was performed using the Review Manager version 5.3. [13] Studies reported a single combined score for knowledge and/or skill with continuous data such as mean and SD or those that were calculated from the available data was considered for the meta-analysis. Comparisons of different types of mobile applications are excluded. Studies with multiple arms and multiple applications are considered as separate studies if their knowledge/skill scores were presented separately. The analyzed data is presented as a standardized mean difference along with SD. All the statistical analyses were performed by one author and cross-checked by another author and any disagreements were resolved by discussion.

#### Heterogeneity and publication bias

We used a random-effect model for the data analysis. Further, the sources of heterogeneity were explored through a subgroup analysis according to the study design. Sensitivity analysis was done to ensure the robustness of the study findings by eliminating studies, which had lesser weight and the least number of participants. Funnel plot asymmetry by visual inspection was used for the detection of publication bias, which was further assessed for statistical significance by Egger’s and Begg’s tests. Stata trial version 15 software was used for the detection of publication bias.

## Results

### Description of studies

#### Results of the search

**Fig 1:**
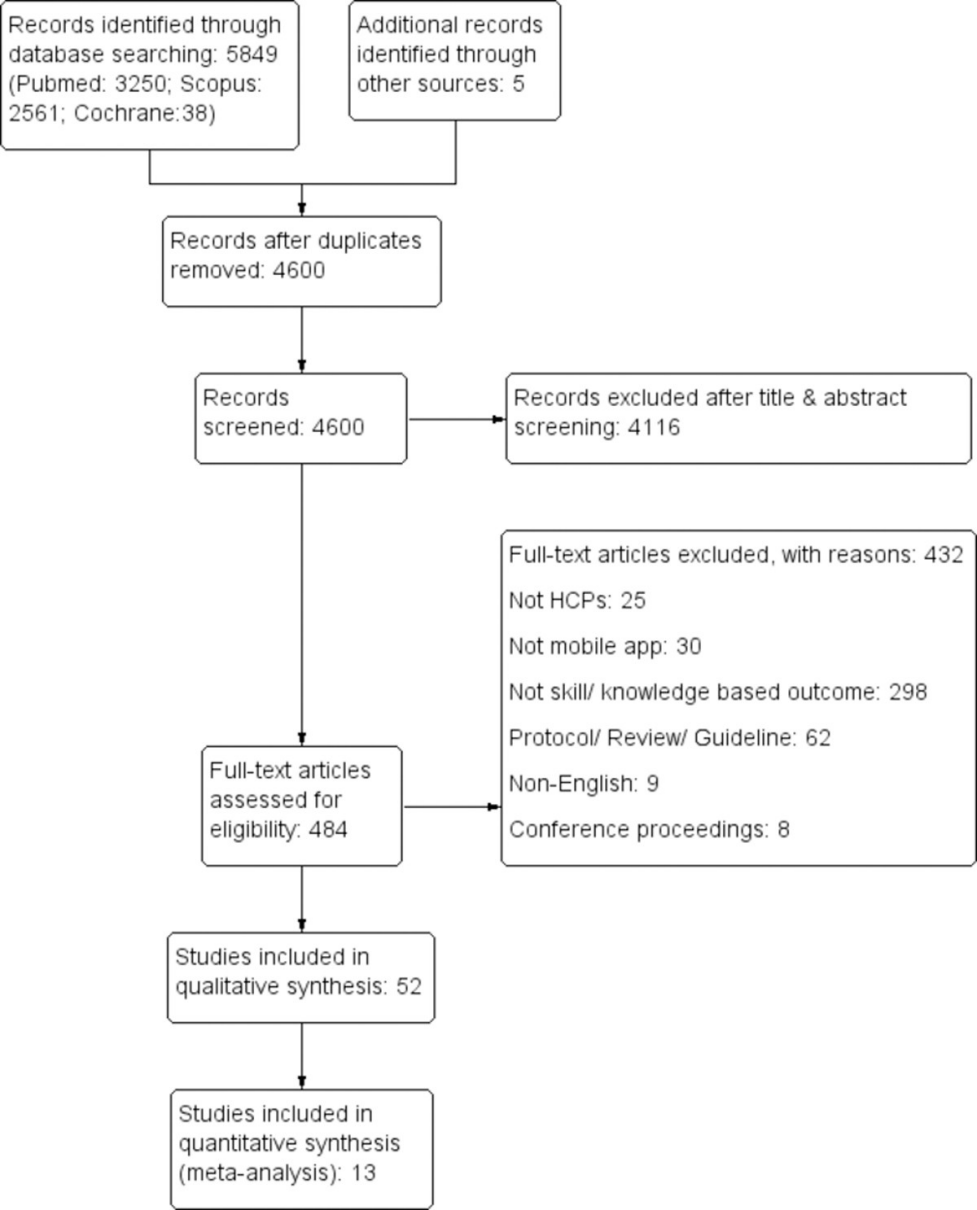
The PRISMA flow diagram.

The literature search identified a total of 5849 records out of which 4600 records were screened after the removal of duplication. Finally, 52 articles were included following the exclusion of 4116 records after the initial screening and 432 articles after the full-text screening. The process of identification, screening, eligibility, and synthesis of findings is illustrated in the PRISMA flow diagram (Fig 1).

#### Summary & characteristics of studies

Out of 52 included studies, 29 assessed change in the knowledge level [8, 14–41], 10 assessed change in the skill level [42–51], and 13 assessed change in both the knowledge and skill level [9, 52–63]. These studies that provided data from a total of 4057 (sample ranges from 5 to 448) learner participants were included in this review. The included studies consist of 27 RCTs, 1 cluster RCT, 8 quasi-experimental studies, 15 interventional cohort studies, and 1 cross-sectional study, which was published between 2011 and 2020. Major portion of the studies (36/52) were performed in developed countries namely USA (n = 10) [18, 21, 23, 24, 32, 34, 38, 42, 50, 60], UK (n = 7) [37, 43, 44, 47–49, 52], South Korea (n = 5) [53, 58–61], Brazil (n = 4) [15, 25, 40, 45], Germany (n = 3) [17, 19, 63], Spain (n = 3) [29, 55, 56], Turkey (n = 2) [22, 54], Chile (n = 2) [9, 51], while the remaining 10 studies were from France [16], Australia [20], China [27], Iran (n = 4) [14, 28, 30, 41], Rwanda [57], India [33], Taiwan [35], Canada [36], Pakistan [8], Thailand [39], Indonesia [26], Palestine [31] and Finland [46]. The duration of the use of mobile applications ranged from onr time use to a year long usage. The characteristics of the included studies are given in the S2 Appendix. It contains study author, country, year, study design, population, sample size, intervention details, outcome measures, and reported limitations in individual studies.

#### Quality of included studies

The quality RCTs and cluster RCT were assessed using the Cochrane risk of bias assessment tool and the results are summarized as the risk of bias graph (S3 Appendix) and summary graph (S4 Appendix). Each category of risk of bias was represented as percentages among the included studies. Interventional and observational studies other than RCTs were assessed using the Modified New castle Ottawa scale. The quality assessment score of included studies using the New castle Ottawa Scale is summarized in the S5 Appendix.

### Effect of intervention on knowledge of HCPs

#### Qualitative analysis

Out of 52 studies, 42 studies reported changes in knowledge level after the use of mobile applications among HCPs/students either in comparison with the control group or comparing the pre and post-test scores of the same group. 33 studies [15 RCTs [9, 14, 15, 17–22, 24–26, 52, 56, 57], 1 cluster RCT [27], 7 quasi-experimental studies [28, 29, 31, 40, 58, 60, 61], 9 interventional cohort studies [33–39, 41, 62] and 1 cross-sectional study [8]] reported that mobile applications were effective tools that led to a significant improvement of knowledge level among the participants. Among that, one RCT conducted by Noll et al., [17] compared the effectiveness of augmented reality mobile application vs. mobile application and concluded that augmented reality mobile application was more effective in increasing the knowledge level than mobile application without augmented reality. 8 studies [5 RCTs [16, 23, 53–55], 1 quasi-experimental study [30], and 2 interventional cohort studies [32, 63]] reported that mobile applications were not effective in improving knowledge among study participants. Among that, one RCT conducted by Kim et al., 2018 [53] compared the effectiveness of interactive mobile application vs non-interactive mobile application and concluded that there is no significant difference in the knowledge level improvement between the two groups. Another quasi-experimental study conducted by Young Yoo et al., [59] reported that mobile applications were effective in improving knowledge in one study condition only (out of the 2 conditions). The overall summary of the effects of mobile applications on the knowledge of HCPs is illustrated in Table 1.

**Table 1:** Effect of Intervention on knowledge level.

Study number	Author, year & country	Outcome of interest	Summary statistics (Percentage of mark/ Score by using mobile application (Mean±SD, P Value))	Sample size (I: Intervention; C: Control)	Duration	Effectiveness of mobile application
1	Bonabi et al, 2019, Iran [14]	Change in knowledge level	**Mean knowledge score**: Cont: Pre test: 8.17 ± 2.03; Post test: 10.43 ± 1.8 (**P<0.001**); Int: Pre test: 7.51 ± 1.7; Post test: 10.7 ± 2.1 (**P<0.001**)	107 (I: 57; C:50); Final evaluation: 86 (I: 43; C:43)	4 months	Effective
2	Velasco et al, 2015, Brazil [15]	Change in knowledge level	**Mean score in the intervention:** Pre test: 4.8±3; Post test: 7.5 ± 2 (**P = 0.000**) **Mean score in the control:**Pre test: 5.9 ± 3; Post test: 7.5±3 (**P = 0.005**)	66 (I: 33; C: 33)	2 weeks	Effective
3	Clavier et al, 2019, France [16]	Change in knowledge level	**Post test scores:** **SCT:** Int: 60±9%; Cont: 68±11%; (**P = 0.006**) **MCQs:** Int: 18±4; Cont: 16±4; (P = 0.22)	62 (I: 32; C: 30); Final evaluation: 44 (I:22; C:22)	3 weeks	Not effective
4	Noll et al, 2017, Germany [17]	Change in knowledge level	**Average improvement in score (Immediately after learning)**: Group A: 3.59±1.48; Group B: 3.86±1.51; (P = 0.1) **After 14 days follow-up:** Average decrease of the number of correct answers as follows; Group A: 0.33±1.62; Group B: 1.14±1.30	44 (Group A:22; Group B: 22)	45 min	Effective
5	Samra et al, 2016, USA [18]	Change in knowledge level	**Range of score in intervention (Out of 16)**: Pre test: 0–1; Post test: 0–12 (**P = 0.01**) **Range of score in control (Out of 16)**: Pre test: 0–2; Post test: 0–4 (P = 0.08); **Average improvement in score**: Int: 5.4 points (range, 0–12 points); Cont: 0.5 points (range, –1 to +1 points) (**P = .0286**)	29 (I: 15; C: 14); Final evaluation 21 (I: 7, C:14)	8 weeks	Effective
6	Albrecht V et al, 2013, Germany [19]	Change in knowledge level	**Difference in Pre-post score:** Int: 4.7±2.9; Cont: 3±1.5 (**P = .03**).	10 (I: 6; C: 4)	105 minutes	Effective
7	Stirling et al, 2014, Australia [20]	Change in knowledge level	**Pre test score:** Int: 9.289 ± 2.265; Cont: 9.727 ±2.565; **Post test score:** Int: 10.737±1.996; Cont: 10.424 ±2.437; **Difference in score:** Int: 1.447, 0.384 (**P = 0.001**); Cont: 2.368, 0.436 (P = 0.12)	71 (C: 33; I: 38)	One practical session	Effective
8	Amer et al, 2017, USA [21]	Change in knowledge level	**The mean grade on the standardized test:** Int: 89.3±6.0%; Cont: 75.6±8.7%; (**P < .05**)	100 (C: 50; I: 50)	3 times visualization	Effective
9	Kucuk et al, 2016, Turkey [22]	Change in knowledge level	**Academic achievement score:** Int: 78.14±16.19; cont: 68.34±12.83 (**P<0.05**)	70 (I: 34; C: 36)	5 hours	Effective
10	Brown et al, 2018, USA [23]	Change in knowledge level	**The increment in mean score:** Int: 34% to 81%; Cont: 33% to 63%; (P = 0.81)	67	4 days	Not effective
11	Lacy et al, 2018, USA [24]	Change in knowledge level	**Mean Pre test score:** Int: 75%; Cont: 74.7%; **Mean post test score:** Int: 86.3%; Cont: 77.5%	36	1 hour	Effective
12	Fernandes Pereira et al, 2016, Brazil [25]	Change in knowledge level	**Mean score (Out of 10):** Int: 8.14±1.67; Cont: 5.02±3.21 **Error Average**: Int: 1.83±0.5; Cont: 4.98±1.0 **Average execution time** (min): Int: 15.7±21; Cont: 38.9±4.3	100 (C: 50; I: 50)	4 months	Effective
13	Putri et al, 2019, Indonesia [26]	Change in knowledge level	**Mean difference in BLS knowledge:** Int: 33.75±12.09; Cont: 25.41±10.93; P = 0.016	48 (I: 24; C: 24)	NA	Effective
14	Martínez et al, 2017, Chile* [9]	Change in knowledge level	**Increase in score:** App group: 16.2 ± 8.3 (**P < 0.001**); Control: 10.6 ± 11.7 (**P < 0.001**) Difference in score between the groups: 3.5 (P = 0.22).	80 (I: 40; C: 40)	4 weeks	Effective
15	Naveed et al, 2018, England [52]	Change in knowledge level	**Average MCQ Score** (Percentage): Int: 62.95±5.37; Cont: 56.73±5.18; **P = 0.0285**	20 (C: 10, I: 10); Final evaluation: 15 (C: 7, I: 8)	Int: 1 hr; Cont: 2 hrs	Effective
16	Kim et al, 2018, South Korea [53]	Change in knowledge level	**Improvement in mean knowledge (Out of 23):** Int: 21.24±1.74 to 22.18±0.76; Cont: 20.84±1.35 to 21.25±1.41 **Mean Difference in Pre-post Knowledge:** Int: 0.94±1.74; Cont: 0.41±1.04; P = 0.133	72 (C: 36; I: 36) Final evaluation: 66 (C: 32, I: 34)	1 week	Not effective
17	Kang et al, 2020, South Korea [61]	Change in knowledge level	**Mean Difference in Pre-post Knowledge**: Cont: 0.02 ± 0.13; Exp 1: 0.15 ± 0.14; Exp 2: 0.11 ± 0.15; P = 0.004	86 (Exp 1: 26; Exp 2: 32; Cont: 28)	2 weeks	Effective
18	Bayram et al, 2019, Turkey [54]	Change in knowledge level	**Median First Knowledge test scores:** Int: 18 (9–22); Cont: 17 (12−23); P = 0.441 **Median Last Knowledge test scores:**Int:19 (13−23); Cont: 19 (8–23); P = 0.568	118 (C: 59; I:59)	1 week	Not effective
19	Fernández-Lao et al, 2016, Spain [55]	Change in knowledge level	**Knowledge test (out of 10 points):** Int:7.21 ± 1.988; Cont: 8.09 ± .921; P = 0.089	49 (I: 25; C: 24)	2 weeks	Not effective
20	Lozano-Lozano et al, 2020, Spain [56]	Change in knowledge level	**Pass percentage (MCQ):** Int: 86% (43/50); Cont: 27% (15/55); **P<0.001**	110 (C: 55, I: 55) Final Evaluation: 105 (C: 55, I: 50)	2 weeks	Effective
21	Bunogerane et al, 2017, Rwanda [57]	Change in knowledge level	**Percentage change in knowledge:** Tendon repair theory: Cont:13.0% (P = 0.535); Int: 39.1% (P = 0.056) Tendon repair technique: Cont:19.0% (P = 0.165); Int: 38.1% (**P = 0.0254**)	27 (C: 13; I: 14)	Till post-test completion	Effective
22	Wang et al, 2017, China [27]	Change in knowledge level	**Base line:** Int: 19.43±2.48; Cont: 19.04± 2.66; **Post test (2**^**nd**^ **week):** Int:24.18 ± 3.51; Cont: 20.02 ±2.53; **Post test (3**^**rd**^ **month):** Int: 23.61 ± 3.37; Cont:19.54 ± 2.67;	115 (I: 61; C: 54)	3 months	Effective
23	Ziabari et al, 2019, Iran [28]	Change in knowledge level	**Increment inmean awareness score**: Int: 11.44 ± 2.37 to 14.88 ± 1.97, **P < 0.0001**; Cont: 11.38 ± 3.22 to 12.54 ± 3.04; **P<0.0001**; **Mean difference in BLS awareness score**: Int: 3.44±1.48; Cont:1.16±1.51; **P<0.0001**	100 (I: 50; C: 50)	3 months	Effective
24	Briz-Ponce et al, 2016, Spain [29]	Change in knowledge level	**Pre–post test scores in intervention:** Pre test: 2.2000±1.3732; Post test: 3.6000±1.12122 (**P = 0.031**) **Pre–post test scores in control:** Pre test: 2.6667±1.54303; Post test: 2.4000±1.40408 (P = 0.157)	30 (I: 15; C: 15)	3 Sessions	Effective
25	Golshah et al, 2020, Iran [30]	Change in knowledge level	**Mean grade point:** Post test: Int: 15.57 ± 0.91; Cont: 15.39 ± 1.09; P = 0.503	53 (I: 27; C: 26)	2 weeks	Not effective
26	Salameh et al, 2020, Palestine [31]	Change in knowledge level	**Mean difference in pre-post test scores:** Int: 3.86±1.65; Cont: 1.5±2.21; P<0.000	104 (I: 52; C: 52)	NA	Effective
27	Kang et al, 2018, South Korea# [58]	Change in knowledge level	**Mean HTN knowledge:** Pre test:Int: 89.4±5.9; Cont: 87.4±7.5; Post test:Int: 93.7±4.4; Cont: 87.4±4.7; **P = 0.001** **Mean DM knowledge:** Pre test:Int: 86.5±8.3; Cont: 84.9±6.8; Post test:Int: 91.0±5.7; Cont: 86.0±8.7; **P = 0.009**	92 [I: 49 (HTN: 21; DM: 28); C: 43 (HTN: 20; DM: 23)]	1 week	Effective
28	Young Yoo et al, 2015, South Korea [59]	Change in knowledge level	**Mean Lung test:** Pre test:Int: 6.7±1.0; Cont: 6.6±2.1 (P = 0.666);Post test:Int:7.4±0.8; Cont: 5.8±1.6 (**P = 0.031**) **Mean Heart test:** Pre test: Int:6.4 ± 1.2; Cont:5.7 ± 1.2 (P = 0.161);Post test: Int: 8.3±1.2; Cont: 8.7 ± 0.9 (P = 0.489)	22 (11 each cross over)	4 weeks	Effective in one condition (Out of 2)
29	Kim et al, 2017, South Korea [60]	Change in knowledge level	**Mean Knowledge:** Pre test: Int: 8.69±1.62; Cont: 9.10±1.57 (P = 0.266); Post test: Int: 11.80±1.32; Cont: 11.84±1.48 (P = 0.899) **Mean Difference in knowledge**: Int: 3.11±1.78; Cont: 2.74±1.86 (P = 0.379)	80 (I: 40; C: 40) Final evaluation: 73 (I: 35; C:38)	1 month	Effective
30	Chung et al, 2018, USA [32]	Change in knowledge level	**Difference in mean pre-post score:** Int: 2.4% [–3.1 to 8]; Cont: 4.8% [0.3–9.4]; P>0.05 **Post score:** Int: 73.8% [69.2–78.4]; Cont: 74.1% [70.3–78.0]; P>0.05	37 (I: 18; C: 19)	4 weeks	Not effective
31	Shore et al, 2018, USA [62]	Change in knowledge level	**Median score of Pre test**: 87 (IQR, 81 to 94); **Median score of Post test**: 100 (IQR, 94 to 100).	53 (single group)	3 weeks	Effective
32	Deshpande et al, 2017, India [33]	Change in knowledge level	**Mean score in SCT:** Pre test: 41.5±1.7; Post test: 63±2.4 (**P < 0.005**).	92 (single group)	1 year	Effective
33	Man et al, 2014, USA [34]	Change in knowledge level	**Mean total score (%):** Percentage of correct answers increased after 12 weeks when compared to baseline (P = 0.205). **Mean confidence level**: Managing outpatient adults with major depression: Baseline: 4.214; After 12 Weeks: 5.364 (**P = 0.048**); Starting an antidepressant for newly diagnosed major depression: Baseline: 4.286; After 12 Weeks: 5.636 (**P = 0.018**); Choosing an antidepressant based on patient factors: Baseline: 3.642; After 12 Weeks: 5.273 (**P = 0.010**)	N = 14 (Single group)	12 weeks	Effective
34	Liu et al, 2018, Taiwan [35]	Change in knowledge level	**Average pre-course score:** Overall: 27.50±15.83; Non-dermatology trainees: 15.38 ±8.03; Dermatology trainees: 39.62 ± 11.81. **Average post course score:** Overall: 91.44 ± 5.92; Non-dermatology trainees: 90.77 ± 5.98; Dermatology trainees: 92.12 ± 6.02	26 (Group 1:13; Group 2: 13)	3 weeks	Effective
35	Fralick et al, 2017, Canada [36]	Change in knowledge level	**Improvement in knowledge score**: Int: 6.2±2.1 vs 8.1±2.2 (**P = 0.0001**); Cont: 7.1±1.7 vs 7.5±2.0 (P = 0.23) **Unadjusted linear regression analysis: P = 0.006** [95% CI: 0.46, 2.48]) **Adjusted multivariable linear regression analysis: P = 0.04** [95% CI: 0.10, 2.1])	62 (I: 32; C: 30); Follow up: 53 (I: 27; C: 26)	4 weeks	Effective
36	Weldon et al, 2019, UK [37]	Change in knowledge level	**Percentage of correct answers**: Pre test: Q1: 40%; Q2: 100%; Q3: 60%; Q4: 20%; Q5: 20%; Q6: 20% Post test: Q1: 80%; Q2: 100%; Q3: 80%; Q4: 60%; Q5: 20%; Q6: 80%	5 (Single group)	Till completion of post test	Effective
37	Smeds et al, 2016, USA [38]	Change in knowledge level	**NBME score**: Int: 77.5%; Cont: 68.8% (**P< 0.01**); **USMLE scores**: Int: 225.4; Cont: 209.8; (**P < 0.001**); **Cumulative GPA**: Int: 3.3; Cont: 2.9; (**P < 0.001**); **Mean MCAT scores:** Int: 9.6; Cont: 8.9; (**P < 0.01**).	288 (I: 152; C: 136)	1 year	Effective
38	Ebner et al, 2019, Germany [63]	Change in knowledge level	**Mean Score of MCQs**: Int: 30.2; Cont: 36.8 (P = 0.13)	66 (I: 33; C: 33)	1 week	Not effective
39	Hirunyanitiwattana et al, 2020, Thailand [39]	Change in knowledge level	**General asthma knowledge scores:** Asthma knowledge in both groups improved significantly between pre & post test (WAAP: P = 0.135; ACA: P = 0.002) **Asthma action plan knowledge scores:** No statistical difference in score between, or within each group	44 (I: 25; C: 19)	3 hours	Effective
40	Baccin et al, 2020, Brazil [40]	Change in knowledge level	**Mean grade:** Pre test: 4.77±1.63; Post test: 8.49±1.27 (P<0.0001).	161 Final evaluation: 150	7 weeks	Effective
41	Ameri et al, 2020, Iran [41]	Change in knowledge level	**Difference in mean pre-post test score:** Cont: 0.06 (7.69 to 7.75; P = 0.84); Group 1: 1.88 (7.71 to 9.59; **P<0.0001**); Group 2: 7.6 (7.5 to 15.1; **P<0.0001**)	316 (C: 106 Group 1: 105 Group 2: 105)	2 months	Effective
42	Hisam et al, 2019, Pakistan [8]	Change in knowledge level	**Score in the last professional examination**: Int: 69±7%; Cont: 67±9%. Average usage of application VS academic performance (**P<0.01**)	448 (I: 323; C: 125)	NA	Effective

ACA: Asthma Care Application; AR: Augmented Reality; BLS: Basic Life Support; BSE: Breast Self Examination; CI: Confidence Interval; DM: Diabetes Mellitus; GPA: Grade Point Average; Hr: Hour; HTN: Hypertension; IQR: Inter Quartile Range; MCAT: Medical College Admissions Test; MCQ: Multiple Choice Questionnaire; MD: Mean Deviation; NA: Not Available; NBME: National Board of Medical Examiners; RCT: Randomized Controlled Trial; SCT: Script Concordance Test; SD: Standard Deviation; SEM: Standard Error of the Mean; UK: United Kingdom; USA: United States of America; USMLE: United States Medical Licensing Examination; WAAP: Written Asthma Action Plan

* Indicates that the study reported final score of EUNACOM (theoretical practical exam of general medicine) as combined knowledge and skill score. So the same result is repeated in skill domain also.

# indicates that the study considered as 2 studies in meta analysis, as it is evaluating 2 apps

#### Quantitative analysis

**Fig 2:**
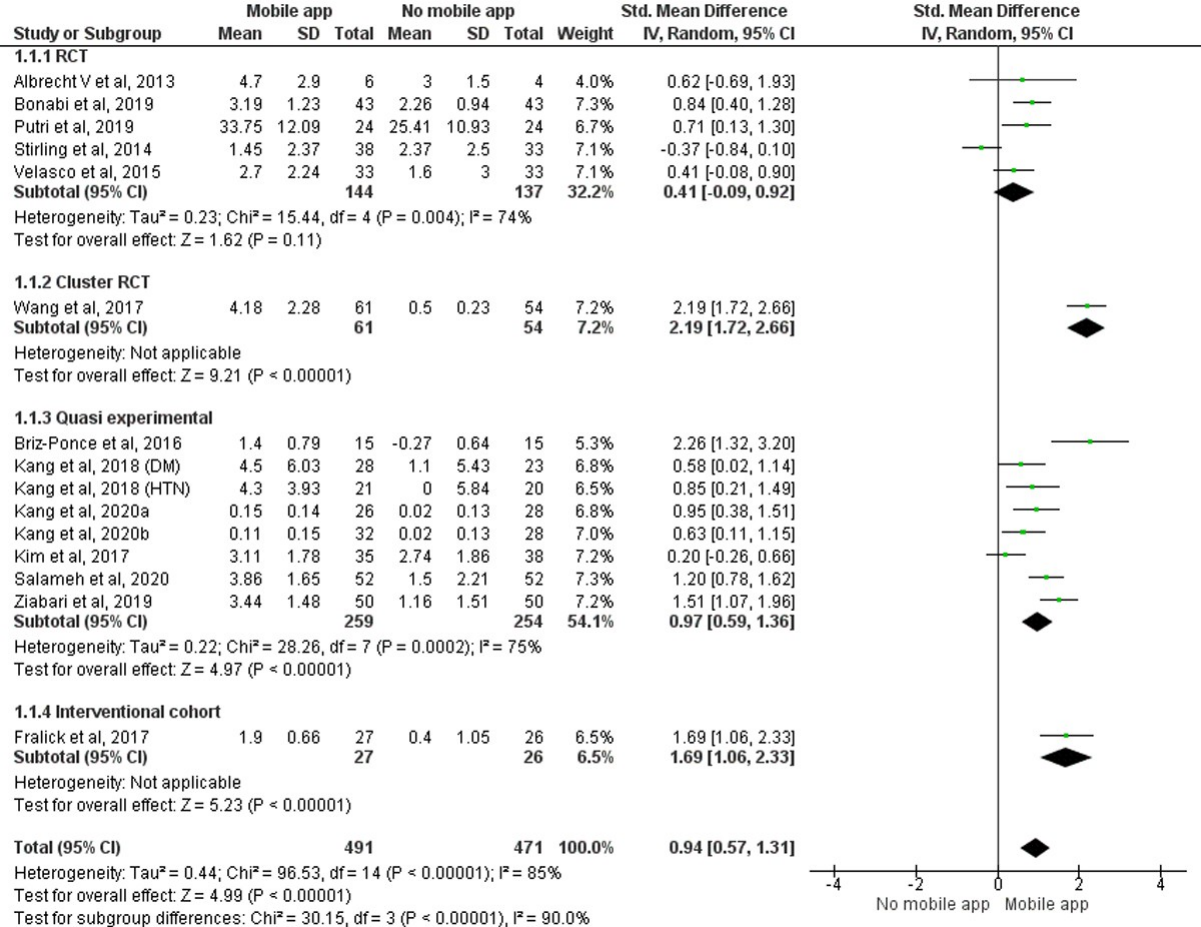
Forest plot: Effect of intervention on knowledge of HCPs.

Pooled effect of 15 [14, 15, 19, 20, 26–29, 31, 36, 58, 60, 61] studies with 962 participants showed that the knowledge score improved significantly in the group using the mobile application when compared to the group that does not use mobile application (SMD = 0.94, 95% CI = 0.57 to1.31, P<0.00001) (Study by Kang 2018 [58] is considered as two studies in the meta-analysis as it analyzes the effectiveness of two different applications and reported the outcomes separately. The study by Kang 2020 [61] is considered as two studies in the meta-analysis as it has three groups and analyzed outcomes separately). The random effect model was considered because of the significant statistical heterogeneity among studies (Fig 2). A subgroup analysis was performed because of the heterogeneity in the included study designs. The pooled effect of individual study designs (Cluster RCT, quasi experimental studies and interventional cohort study) showed that participants using mobile applications had better knowledge enhancement scores compared to the group that did not use mobile applications among the study participants (Cluster RCT: SMD = 2.19, 95% CI = 1.72 to2.66, P<0.00001; quasi-experimental studies: SMD = 0.97, 95% CI = 0.59 to 1.36, P<0.00001; interventional cohort study: SMD = 1.69, 95% CI = 1.06 to 2.33, P <0.00001) giving total weightage of 67.8%.

### Effect of intervention on skills of HCPs

#### Qualitative analysis

Out of 52 studies, 23 studies reported changes in skill level after the use of mobile applications among healthcare professionals/students either in comparison with the control group or comparing the pre and post-test scores of the same group. 19 studies [11 RCTs [9, 43–45, 47, 48, 52, 53, 55–57], 3 quasi-experimental studies [58, 60, 61] and 5 interventional cohort studies [49–51, 62, 63]] reported that mobile applications were effective tool and significantly improved skill levels among participants. Among that, one RCT conducted by Kim et al., in 2018 [53] compared the effectiveness of an interactive mobile application vs a non-interactive mobile application and concluded that interactive mobile application was more effective in improving skill than the non-interactive mobile application. Three studies [2 RCTs [46, 54] and 1 quasi-experimental study [59]] reported that mobile applications were not effective in improving skill among study participants. One RCT conducted by Nadir et al., in 2019 [42] reported that a mobile application was effective in improving skill in one study condition only (out of two conditions). The overall summary of the effects of mobile applications on the knowledge of HCPs is illustrated in Table 2.

**Table 2:** Effect of Intervention on skill level.

Study number	Author, year & country	Outcome of interest	Summary statistics (Percentage of mark/ Score by using mobile application (Mean±SD, P Value))	Sample size (I: Intervention; C: Control)	Duration	Effectiveness of mobile application
1	Nadir et al, 2019, USA [42]	Change in skill level	**Mean percentages of completed checklist items**: Int: 56.1±10.3; Cont: 49.4±7.4 for shortness of breath (**P = 0.001**); Int: 58±8.1; Cont: 49.8±7.0 for syncope (**P<0.001**) **Mean GRS score for Syncope:** Int:3.14±0.89; Cont: 2.6±0.97; **P = 0.003** **Mean GRS score for shortness of breath:** Int:2.90±0.97; Cont: 2.81±0.80; P = 0.43	58 (C:29; I: 29)	23 minutes	Effective in one condition (Out of 2)
2	Mamtora et al, 2018, UK [43]	Change in skill level	**Accurate description score (Out of 60):** Dry AMD: Int: 47(78%); Cont: 28(47%) **P < 0.05**; CRVO: Int: 31(52%); Cont: 15(25%) **P < 0.05**; Papilloedema: Int: 28(47%); Cont: 29(48%) p = 0.52; Optic atrophy: Int: 50(83%); Cont: 34(57%); P = 0.08; PDR: Int: 43(72%); Cont: 32(53%); **p = < 0.05** **Accurate clinical diagnosis score (Out of 20):** AMD: Int: 15(75%); Cont: 6(30%) **P < 0.05**; CRVO: Int: 8(40%); Cont: 6(30%); P = 0.48; Papilloedema: Int: 6(30%); Cont: 6(30%); P = 0.78; Optic atrophy: Int: 4(20%); Cont: 4(20%) P = 0.66; PDR: Int: 14(70%); Cont: 8(40%) P = 0.10	20 (C: 10; I: 10)	15 min	Effective
3	Haubruck et al, 2018, UK [44]	Change in skill level	**Operation performance by OSATS (Points)**: Int: 38.0 [I_50_ = 7.0]; Cont: 30.5 [I_50_ = 8.0]; (**P<0.001**); **Economy of time and motion:** Int: 4.0 [I_50_ = 1.0]; Cont: 3.0 [I_50_ = 1.0]; (**P = 0.004**); **Less helping need:** Int: 4.0 [I_50_ = 1.0]; Cont: 2.0 [I_50_ = 1.0]; (**P<0.001**); **Confident in handling of instruments:** Int: 3.0 [I_50_ = 2.0]; Cont: 3.0 [I_50_ = 2.0] (**P<0.001**); **Digital exploration of the pleural cavity**: Int: 4.0 [I_50_ = 2.0]; Cont: 2.0 [I_50_ = 2.0](**P<0.001**); **Median time of performing a CTI:** Int: 4:15; Cont: 4:17 min.	95 (I: 49; C: 46)	120 min	Effective
4	Oliveira et al, 2019, Brazil [45]	Change in skill level	**Significant differences from the specialists’ reference standards:** Int: Conditions 4 and 10 (**P<0.001**); Cont: Conditions 1, 4,6, 7, 8, and 10 (**P<0.05**)	20 (C: 10; I: 10)	1 month	Effective
5	Martínez et al, 2017, Chile* [9]	Change in skill level	**Increase in score:** Int: 16.2 ± 8.3 points (**P < 0.001**); Cont: 10.6 ± 11.7 points (**P < 0.001**) Difference in score between the groups: 3.5points (P = 0.22).	80 (I: 40; C: 40)	4 weeks	Effective
6	Naveed et al, 2018, England [52]	Change in skill level	**Average OSATS Score** (Max score: 5): Int: 3.53±0.39; Cont: 2.58±0.71; **P = 0.0139**	20 (C: 10; I: 10); Final evaluation (C: 7; I: 8)	Int: 1 hour; Cont: 2 hours	Effective
7	Kim et al, 2018, South Korea [53]	Change in skill level	**Improvement in nursing skill performance (Out of 378)**: Int: 205.35 ± 24.01 to 363.62 ± 9.07; Cont: 202.94 ± 22.95 to 328.22 ± 27.76 **Mean Difference in Pre-post Skills:** Int: 158.26±25.61; Cont: 125.28±33.502; **P<0.001**	72 (C: 36; I: 36); Final evaluation: 66 (C: 32, I: 34)	1 week	Effective
8	Kang et al, 2020, South Korea [61]	Change in skill level	**Mean Difference in Pre-post Skills:** Cont: 0.52 ± 0.56; Exp 1: 0.62 ± 0.73; Exp 2: 0.95 ± 0.46; P = 0.014	86 (Exp 1: 26; Exp 2: 32; Cont: 28)	2 weeks	Effective
9	Bayram et al, 2019, Turkey [54]	Change in skill level	**Median Skill score:** Pre test:Int: 53 (40–57); Cont: 53 (37–57); P = 0.997 Post test:Int: 55 (46–57); Cont: 54 (46–57); **P = 0.017** **Median OSCE time (Seconds):** Pre test: Int: 330 (162–360); Cont: 340 (228–360); P = 0.022 Post test:Int: 260 (180–360); Cont: 260 (190–360); P = 0.723	118 (C: 59; I:59)	1 week	Not effective
10	Fernández-Lao et al, 2016, Spain [55]	Change in skill level	**Global OSCE Scores:** **Ultrasound skills**: Int:12.000 ± 2.572; Cont:9.000 ± 2.943; **P = 0.000** **Palpation skills**: 12.038 ± 3.155; Cont:9.833 ± 3.963; **P = 0.034**	49 (I: 25; C: 24)	2 weeks	Effective
11	Lozano-Lozano et al, 2020, Spain [56]	Change in skill level	**OSCE exam score:** Int:7.3 ± 1.5; Cont: NA; **P<0.001**	110 (C: 55; I: 55); Final Evaluation: 105 (C: 55; I: 50)	2 weeks	Effective
12	Strandell-Laine et al, 2018, Finland [46]	Change in skill level	**Mean Overall improvement in competence score**: Int: 10.11±2.22; Cont: 11.67±2.30 (P = 0.57) **Mean Improvement in self-efficacy:** Int: 1.77±0.17; Cont: 1.51± 0.20 (P = 0.37)	102 (I = 52; C = 50)	Three periods of 5 weeks	Not effective
13	Bartlett et al, 2017, UK [47]	Change in skill level	**Mean baseline score to post intervention score:** Group 1: 28.7 (62.3%) to 32.7 (71.1%); P = 0.003 Group 2: 27.0 (58.7%) to 36.1 (78.5%); P = 0.001 Group 3: 27.6 (59.8%) to 34.9 (75.7%); P = 0.001 **Mean score change from baseline (95% CI):** Group 1: 4.0 (1.8–6.2); Group 2:9.1 (4.7–13.5); Group 3: 7.3 (4.3–10.4)	27 (Group 1: 9; Group 2: 9; Group 3: 9)	1 hour	Effective
14	Bunogerane et al, 2017, Rwanda [57]	Change in skill level	**Difference in cognitive skills (percentage change in pre test & post test):** Int: 38.6% (P<0.001); Cont: 15.9% (P = 0.304) **Overall simulation test score:** Int: 22.43 (89.71%); Cont: 15.85 (63.4%); P <0.001	27 (C: 13; I: 14)	Till post-test completion	Effective
15	Low et al, 2011, UK [48]	Change in skill level	**Overall cardiac arrest simulation test score Median (IQR):** Int: 84.5(75.5–92.5); Cont: 72(62–87); **P = 0.02**	31 (I: 16; C: 15)	Till completion of test	Effective
16	Miriam McMullan, 2018, UK [49]	Change in skill level	**Drug Calculation Ability (Mean**±**SD)**: Pre: 47.6±23.4; Post: 56.7±24.7; **P = 0.004** **Drug Calculation Self-Efficacy (Mean**±**SD)**: Pre: 20.4±18.0; Post: 49.6±19.9; **P<0.001**	60 (Paramedics: 41; ODP: 19)	8 weeks	Effective
17	Kang et al, 2018, South Korea [58]	Change in skill level	**Mean HTN self-efficacy** **Pre test:** Int:72.2±9.6;Cont:68.1±13.6; **Post test:** Int:78.0±10.3;Cont:66.4±13.6; **P = 0 .002** **Mean DM self-efficacy** **Pre test:** Int:67.8±9.9;Cont: 62.2±14.1; **Post test:** Int:72.0±12.2;Cont: 65.8±12.6; **P = 0.043**	92 [I: 49 (HTN: 21; DM: 28); C: 43 (HTN: 20; DM: 23)]	1 week	Effective
18	Young Yoo et al, 2015, South Korea [59]	Change in skill level	**Clinical assessment skill for lung practice:**Int:29.0 ± 1.1; Cont: 28.4 ± 0.8 (P = 0.258) **Clinical assessment skill for Heart practice:** Int: 37.0 ± 2.4; Cont: 38.3 ± 1.3 (P = 0.258)	22 (11 each cross over)	4 weeks	Not effective
19	Kim et al, 2017, South Korea [60]	Change in skill level	**Difference in the mean scores for skills:** Int: 11.97 ± 5.07; Cont: 6.71 ± 4.34 (**P<0.001**)	80 (I: 40; C: 40); Final evaluation: 73 (I: 35; C:38)	1 month	Effective
20	Shore et al, 2018, USA [62]	Change in skill level	**Median simulation test score:** 89 (IQR, 81 to 92)	53 (single group)	3 weeks	Effective
21	Meyer et al, 2018, USA [50]	Change in skill level	**Mean accuracy in testing/ diagnostic decisions:** Int: 82.6%; Cont: 70.2%; **P<0.001** **Mean confidence in testing/diagnostic decisions (out of 10):** Int: 7.5; Cont: 6.3; **P<0.001**	46	30 to 60 minutes	Effective
22	Quezada et al, 2019, Chile [51]	Change in skill level	**Improvement in GRS (5–25) score:** Int: 15(6–17) to 23(20–25); **P<0.05**; Cont: 15(10–19) to 24(22–5); **P<0.05** **Improvement in SRS (4–20) score:** Int: 12(11–15) to 18(15–20); **P<0.05**; Cont: 12(8–15) to 19(16–20); **P<0.05** **Change in Operative time (min):** Int: 39(10.47) to 22(3.37); **P<0.05**; Cont: 42(12.58) to 22(3.35); **P <0.05**	55 (C: 25; I: 30)	72 hours for single video	Effective
23	Ebner et al, 2019, Germany [63]	Change in skill level	**Longitudinal kidney measurements (mm)** **Right kidney (Median[IQR]**): Int: 105.3(86.1 to 127.1); Cont: 92(50.4 to 112.2); **P<0.001** **Left kidney (Median[IQR]):**Int: 100.3(81.7 to 118.6); Cont: 85.3(48.3 to 113.4); **P<0.001** **Median Measuring time (in seconds):** Int: 351 (155–563); Cont: 302 (103–527) P = 0.26	66 (I: 33; C: 33)	1 week	Effective

AMD: Age-related Macular Degeneration; CRVO: Central retinal vein occlusion; DM: Diabetes Mellitus; GRS: Global rating scale; HTN: Hypertension; ODP: Operating Department Practice; OSATS: Objective Structured Assessment of Technical Skills; OSCE: Objective structured clinical evaluation; PDR: Pre-proliferative diabetic retinopathy; RCT: Randomized controlled trial; SD: Standard Deviation; SRS: Specific rating scale; UK: United Kingdom; USA: United States of America.

* Indicates that the study reported final score of EUNACOM (theoretical practical exam of general medicine) as combined knowledge and skill score. So the same result is repeated in skill domain also

#### Quantitative analysis

**Fig 3:**
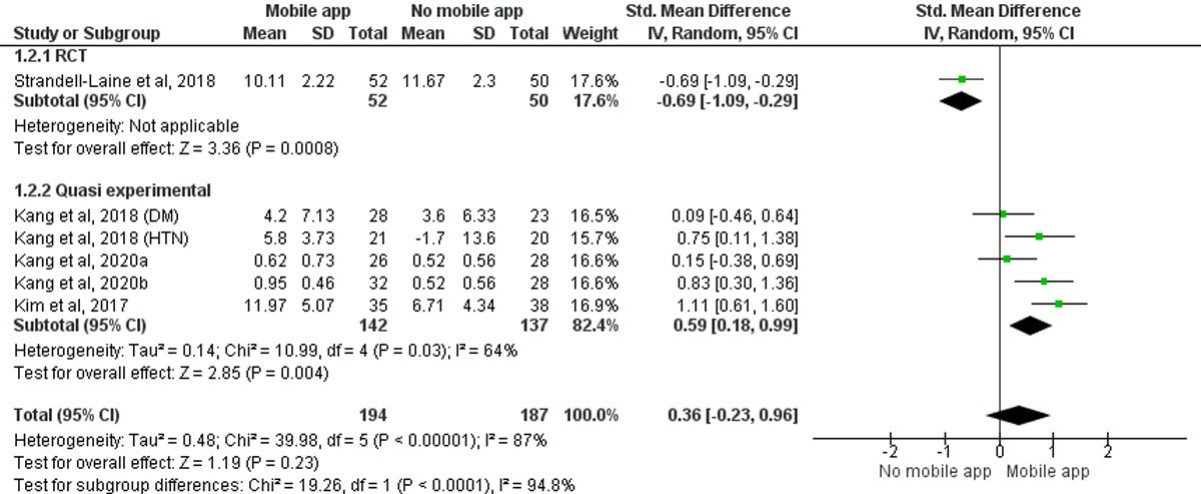
Forest plot: Effect of intervention on the skill of HCPs.

A meta-analysis of six [46, 58, 60, 61] studies with 381 participants studies showed no significant change in skill level among the group using the mobile applications and the group that did not use mobile applications (SMD = 0.36, 95% CI = -0.23 to 0.96, P = 0.23). Included study designs for data synthesis were RCT (n = 1) [46] and quasi-experimental studies (n = 5) [58, 60] (Study by Kang 2018 [58] is considered as two studies in the meta-analysis as it analyzed the effectiveness of two different applications and reported outcomes separately. The study by Kang 2020 [61] is considered as two studies in the meta-analysis as it has three groups and analyzed outcomes separately). The random-effect model was considered because of the significant statistical heterogeneity among studies. A subgroup analysis was performed based on study designs. The pooled effect of quasi-experimental studies showed that the group using the mobile application was better at skill enhancement than the group that did not use mobile application among the study participants (SMD = 0.59, 95% CI = 0.18 to 0.99, P = 0.04) giving a total weightage of 82.4% (Fig 3).

#### Sensitivity analysis

A sensitivity analysis was performed by removing four studies from the knowledge assessment and one study from the skill assessment that has less weightage, which also supported the previous findings. The result of sensitivity analysis is depicted in S6 Appendix and S7 Appendix.

#### Publication bias

An obvious asymmetry was observed by the visual inspection of the funnel plot (S8 Appendix), which was not statistically confirmed by Egger’s (P = 0.5) and Begg’s test (P = 0.49). Hence there is no publication bias among the included studies in the meta-analysis.

#### Educational area of mobile applications

**Fig 4:**
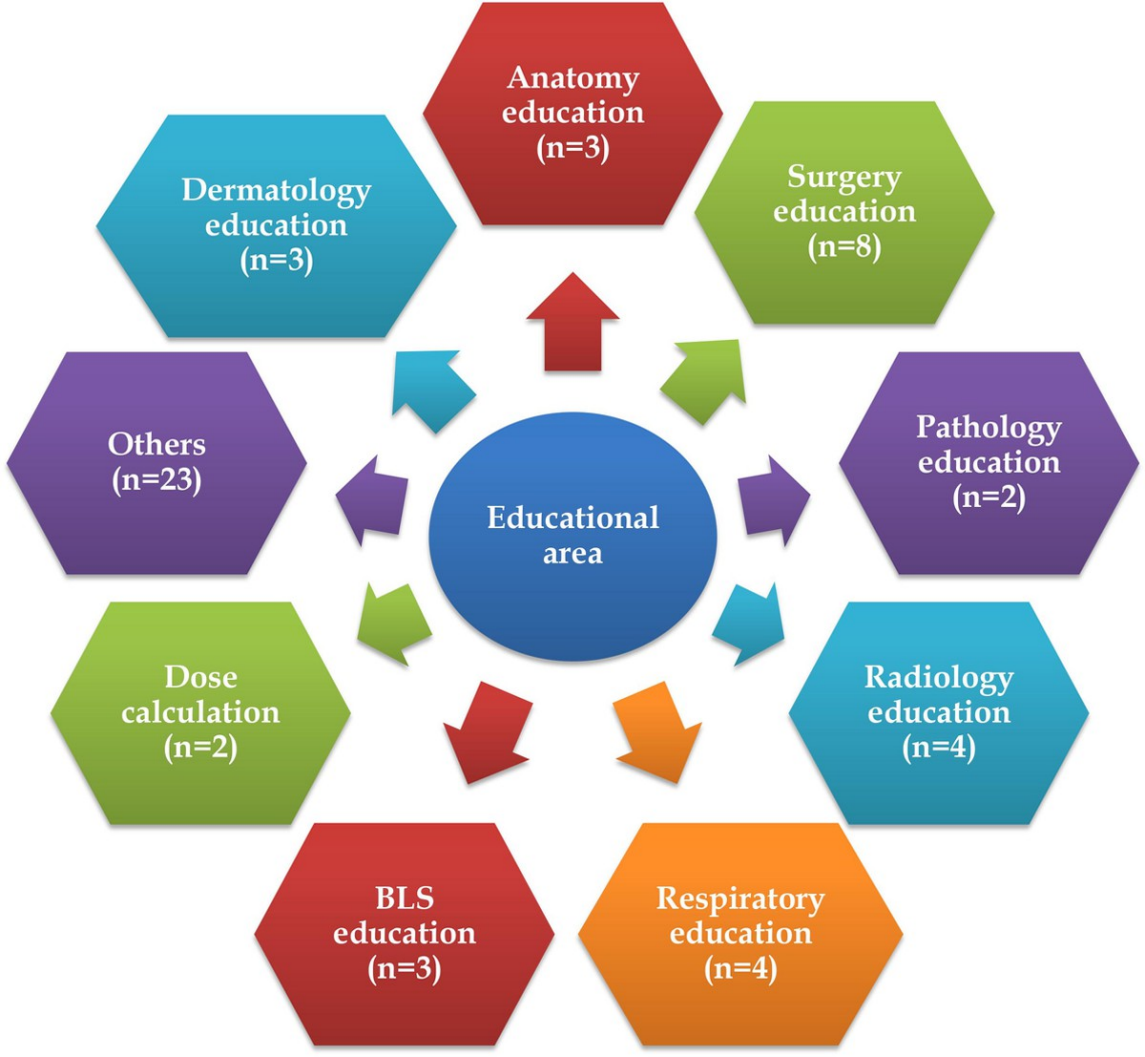
Educational area of mobile applications.

The majority of researchers used mobile applications for education on anatomy, surgery, respiratory conditions, dermatology, basic life support (BLS), pathology, dose calculation, and radiology. The characteristic details of the intervention and educational area details are depicted in S2 Appendix and Fig 4.

### Network connectivity and mobile apps

Most of the applications that cater to medical education were web-based mobile applications that require an internet connection for access. There was no significant change in the enhancement of knowledge and/or skill among participants using online and offline versions of mobile apps (S9 Appendix). Developing mobile applications which can be accessed without internet connectivity will increase the acceptability and use of mobile apps among healthcare professionals and/or students living in low to middle-income countries and rural areas.

### Mobile application operating system

Android and iPhone operating systems (iOS) were used for mobile applications. Both operating system-based applications were effective in enhancing knowledge or skill. There was no significant change in the enhancement of knowledge or skill among participants who used android or iOS mobile apps (S10 Appendix). Developing mobile applications which can be operated through android will increase the acceptability and use of mobile apps among healthcare professionals and/or students living in low to middle-income countries.

## Discussion

This study attempted to evaluate the effectiveness of mobile applications in enhancing knowledge and/or skill among HCPs and/or students. The findings from the study support the claim that mobile applications are an effective tool. Medical, paramedical, and allied healthcare professionals were the target population for evaluating the effectiveness of mobile apps in enhancing knowledge and skills. The educational topic, duration of intervention, nature of control/comparison group, and features of mobile apps were different in different studies. Even if the population and mobile apps have their differences, every study compared the change in knowledge and/or skill quantitatively with or without a comparison group. Variations were present in providing the type of educational material to the control/comparison group in different studies. Studies compared the effectiveness of mobile app learning vs traditional teaching or teaching using audio-video slides, simulator training, hardcopy learning materials and e-learning through computer platforms. All the studies compared the change in knowledge and/or skill level by using a mobile application with any of the regular teaching methods, which did not affect the quality of study results. The meta-analysis of the change in the skill of the included studies was not statistically significant. This is possibly due to the limited number of studies in the meta-analysis. Studies that reported skill as a single comparable score were only considered for the meta-analysis. The majority of studies reported multiple component scores in evaluating the skill of healthcare professionals. The systematic review findings supported the use of mobile applications in the enhancement of skill.

Different studies used different mobile applications with different educational topics. The “Touch surgery” application was used in five studies [21, 44, 47, 57, 62] but the educational topic was different. The choice of topics in a mobile app may differ due to the variety of study population, area of interest/expertise of study coordinators and/or study participants and required facilities (e. g.: simulation) in the institution.

Studies among various healthcare professionals reported mixed results regarding the usefulness of the e-learning, mobile learning and technology-enhanced learning. A Cochrane systematic review conducted by Vaona et al in 2018 compared traditional learning with e-learning and reported that e-learning may make little or no difference in health professionals’ behaviours, skills or knowledge [64]. A study conducted by Subhash et al, 2015 among medical students reported that smartphones can be effectively used for learning [65]. A study conducted by Snashall et al, 2016 among medical students reported that medical apps can be used as an adjunct in medical education, though the evidence remains limited [2]. A qualitative systematic review by the Digital Health Education Collaboration conducted by Lall et al, 2019 on the implementation of mobile learning for medical and nursing education reported that mobile learning can potentially play a substantial role in learning [66]. A study conducted by Dickinson et al, 2020 on the educational applications for surgery residents reported that technology-enhanced learning has become prevalent in surgical education and future studies are needed to assess the efficacy of educational apps for surgical education [67]. By considering all the other published studies to the best of the authors’ knowledge, this is the first systematic review that has attempted to quantitatively analyze the change in knowledge and/or skills by using any mobile application.

Additionally, this is the first systematic review assessing the effectiveness of online vs offline and android vs iOS based mobile applications in enhancing knowledge and/or skill quantitatively. Even if more studies focused on online applications, offline applications also were equally effective. Either android or iOS based mobile applications were used in different studies. Offline mobile applications, which can be accessed through android and iOS will increase the wide acceptability of mobile apps and reduce the economic burden. Moreover, they help tackle problems due to poor internet connectivity. It is the latest growing trend and developers of mobile apps should take tremendous interest in creating these applications [68].

No restrictions were imposed in the publication period during the literature search. But the included studies were published between 2011 and 2020. During the first decade of the 2000s, m-learning had grown in different forms and directions [69]. This may be the reason for the publication period ranging from 2011 to 2020. Among the 52 included studies around 36 studies are from developed nations. The remaining studies are only developing countries. This may be because the concept of m–learning originated in Europe and this encouraged researchers and educators to reconsider their view on mobile technology as a pedagogical tool [70]. Other possible reasons may include lack of resources, fear of adopting mobile technology, lack of skills of instructors, interruption in power supply and internet connectivity, affordability issues, low bandwidth, and trust deficit. This could impact the global representativeness of the review findings. Therefore, future research should address the effectiveness of mobile applications in learning in diverse populations from other countries.

Cochrane risk of bias assessment tool was used to assess the quality of included RCTs. The quality of the majority of included studies in all sub-domains was high to moderate except for the blinding of participants and personnel, which showed a high risk of bias based on the Cochrane tool. It is not possible to blind the participants in the study population as it provides a mobile application for education to the intervention group. Hence the overall risk of bias in the included studies can be considered as low to moderate. Modified New castle Ottawa scale was used to assess the risk of bias in the included studies other than RCTs. All studies have a comparison group scored 4 or more. Hence these can be considered as moderate to high-quality studies. Single group studies can be considered as low-quality studies based on lesser scores in Modified New castle Ottawa scale scoring. The findings from this review can be considered before developing any mobile application in medical education as the results are interpreted from high-quality studies.

However, this study has some limitations. In this study, the search was conducted in three databases only and this may lead to the missing of studies. We tried to avoid the biases due to the missing of studies by selecting large databases such as PubMed, Cochrane library and Scopus and also by developing a comprehensive search strategy by collecting maximum keywords from the published studies and MeSH term search. According to Cochrane guidelines, a minimum of two large electronic databases should be searched. We have chosen 3 major scientific databases for searching studies. Additionally, we have searched the bibliography of included studies, related reviews, and free search in Google Scholar. This resulted in the identification of five more studies. Hence the chance of missing studies is limited in our review. Secondly, we have excluded studies published in other languages except for English, conference proceedings and grey literature. This may lead to the missing of studies published in non-English languages. However, excluding studies from conference proceedings and grey literature sources may increase the strength of study findings by avoiding irrelevant and incomplete data. Heterogeneity in the assessment of knowledge and/or skill of the study population was another limitation. We have included studies that reported quantitative measurement of knowledge and/or skill score to avoid biases due to various measurement approaches. The meta-analysis was performed for studies provided mean and SD or could be calculated from the reported measures only.

### Future directions

Smartphone learning encourages conversation, communication, and cooperation, as well as increased participation. Millennial learners prefer participant-centered, active, and self-directed. Mobile applications based learning can be integrated into medical education to evaluate its performance. Learning materials should be available in a digital format to be feasible to fuel smartphone learning and online education. The importance of education and training using mobile applications is enhanced in the current COVID-19 scenario. A faculty-guided strategy to select appropriate and cost-effective medical education applications can be used. Universities or healthcare organizations should adopt policies for faculty, staff and/or students regarding the use of mobile applications for educational purposes. Multicenter randomized controlled trials with a longer duration have to be conducted to assess the effectiveness and the retention of knowledge and/or skills of mobile applications in medical education among HCPs and/or students. Necessary measures should be employed to avoid dropout, cross-communication between participants and software compatibility issues among trial participants. Other validated practical methods should be employed to assess the knowledge and/or skill validated questionnaires.

## Conclusion

Mobile applications are effective tools in enhancing knowledge and skills among healthcare professionals. Online/offline and android/iOS based applications were equally effective in enhancing knowledge. The prevailing pandemic situation demands medical education to increasingly utilize the opportunities of e-learning instead of conventional teaching. Mobile applications can be considered as an effective adjunct tool in medical education by considering the low expense, high versatility, reduced dependency on regional or site boundaries, online, offline, simulation and learning wherever features of mobile apps.

## Supporting information

S1 Appendix The detailed search strategy in different databases. (DOCX)

S2 Appendix The characteristics of included studies. (DOCX)

S3 Appendix Cochrane risk of bias graph of included RCTs. (PNG)

S4 Appendix Cochrane risk of bias summary of included RCTs. (PNG)

S5 Appendix New castle ottawa score of included studies. (DOCX)

S6 Appendix Sensitivity analysis: Effect of intervention on knowledge of HCPs. (PNG)

S7 Appendix Sensitivity analysis: Effect of intervention on the skill levels of HCPs. (PNG)

S8 Appendix Funnel plot. (PNG)

S9 Appendix Network connectivity and mobile applications. (JPG)

S10 Appendix Mobile application operating system. (DOCX)
