# Supplementary material for: Mobile applications in medical education: A systematic review and meta-analysis
Source: PLoS One. 2022 Mar 24;17(3):e0265927. doi: 10.1371/journal.pone.0265927 (PMC8947018; doi:10.1371/journal.pone.0265927)
Supplement: S1 Appendix — (DOCX) [file pone.0265927.s001.docx]

**S1 Appendix: The detailed search strategy in different databases**

1. PubMed Search strategy

| **Search** | **Query** | **Items found** |
| --- | --- | --- |
| #1 | Search (((((((((((((((((Medical Staff) OR Hospital Medical Staff) OR Hospital Medical Staffs) OR Staff, Hospital Medical) OR Staffs, Hospital Medical) OR Medical Staffs, Hospital) OR Physicians, Junior) OR Junior Physician) OR Junior Physicians) OR Physician, Junior) OR Registrars, Hospital) OR Hospital Registrar) OR Hospital Registrars) OR Registrar, Hospital) OR Attending Physicians, Hospital) OR Hospital Attending Physicians) OR Hospital Attending Physician) OR Attending Physician, Hospital | 116074 |
| #2 | Search ((((((((((((((((((((((((((((((((((Physician) OR Barber Surgeons) OR Allergists) OR Anesthesiologists) OR Cardiologists) OR Dermatologists) OR Endocrinologists) OR Foreign Medical Graduates) OR Gastroenterologists) OR General Practitioners) OR Geriatricians) OR Hospitalists) OR Surgeons) OR Nephrologists) OR Neurologists) OR Occupational Health Physicians) OR Oncologists) OR Radiation Oncologists) OR Ophthalmologists) OR Osteopathic Physicians) OR Otolaryngologists) OR Pathologists) OR Pediatricians) OR Neonatologists) OR Physiatrists) OR Rheumatologists) OR Physicians, Family) OR Physicians, Primary Care) OR Physicians, Women) OR Pulmonologists) OR Radiologists) OR Radiation Oncologists) OR Neurosurgeons) OR Orthopedic Surgeons) OR Urologists | 1107793 |
| #3 | Search (((((Pharmacist) OR Pharmacist, Clinical) OR Clinical Pharmacists) OR Clinical Pharmacist) OR Pharmacists, Clinical) OR Hospital Pharmacist | 40827 |
| #4 | Search (((((((Nurses) OR Nurse) OR Personnel, Nursing) OR Nursing Personnel) OR Registered Nurses) OR Nurse, Registered) OR Nurses, Registered) OR Registered Nurse | 456039 |
| #5 | Search ((((((((((((((((((((((((((((Allied Health) OR Health Personnel, Allied) OR Personnel, Allied Health) OR Allied Health Professional) OR Allied Health Professionals) OR Health Professional, Allied) OR Health Professionals, Allied) OR Professional, Allied Health) OR Professionals, Allied Health) OR Healthcare Assistants) OR Assistant, Healthcare) OR Assistants, Healthcare) OR Healthcare Assistant) OR Healthcare Support Workers) OR Healthcare Support Worker) OR Support Worker, Healthcare) OR Support Workers, Healthcare) OR Worker, Healthcare Support) OR Workers, Healthcare Support) OR Paramedics) OR Paramedic) OR Paramedical Personnel) OR Personnel, Paramedical) OR Population Program Specialists) OR Population Program Specialist) OR Program Specialist, Population) OR Program Specialists, Population) OR Specialist, Population Program) OR Specialists, Population Program | 105663 |
| #6 | Search ((((((((((((((((((Medical Students) OR Student, Medical) OR Medical Student Pharmacy Students) OR Pharmacy Students) OR Student, Pharmacy) OR Pharmacy Student Nursing students) OR Pupil Nurses) OR Student, Nursing) OR Nurses, Pupil) OR Nurse, Pupil) OR Pupil Nurse) OR Nursing Student) OR Nursing Students) OR Premedical Students) OR Student, Premedical) OR Premedical Student) OR Pharmacy Students) OR Student, Pharmacy) OR Pharmacy Student | 185684 |
| #7 | Search (#1 OR #2 OR #3 OR #4 OR #5 OR #6) | 1784412 |
| #8 | Search (((((((((((((((((((((((((((((((Mobile application) OR Application, Mobile) OR Applications, Mobile) OR Mobile Application) OR Mobile Apps) OR App, Mobile) OR Apps, Mobile) OR Mobile App) OR Portable Electronic Apps) OR App, Portable Electronic) OR Apps, Portable Electronic) OR Electronic App, Portable) OR Electronic Apps, Portable) OR Portable Electronic App) OR Portable Electronic Applications) OR Application, Portable Electronic) OR Applications, Portable Electronic) OR Electronic Application, Portable) OR Electronic Applications, Portable) OR Portable Electronic Application) OR Portable Software Apps) OR App, Portable Software) OR Apps, Portable Software) OR Portable Software App) OR Software App, Portable) OR Software Apps, Portable) OR Portable Software Applications) OR Application, Portable Software) OR Applications, Portable Software) OR Portable Software Application) OR Software Application, Portable) OR Software Applications, Portable | 25598 |
| #9 | Search (#7) AND #8 | 3250 |

1. SCOPUS Search Strategy

| **Search** | **Query** | **Items found** |
| --- | --- | --- |
| #1 | TITLE-ABS-KEY ("Medical Staff" OR "Hospital Medical Staff" OR "Hospital Medical Staffs" OR "Staff Hospital Medical" OR "Staffs Hospital Medical" OR "Medical Staffs Hospital" OR "Physicians Junior" OR "Junior Physician" OR "Junior Physicians" OR "Physician Junior") | 55442 |
| #2 | TITLE-ABS-KEY ("Physician" OR "Barber Surgeons" OR "Allergists" OR "Anesthesiologists" OR "Cardiologists" OR "Dermatologists" OR "Endocrinologists" OR "Foreign Medical Graduates" OR "Gastroenterologists" OR "General Practitioners" ) | 922991 |
| #3 | TITLE-ABS-KEY ("Allied Health" OR "Health Personnel Allied" OR "Personnel Allied Health" OR "Allied Health Professional" OR "Allied Health Professionals" OR "Health Professional Allied" OR "Health Professionals Allied" OR "Professional Allied Health" OR "Paramedics" ) | 27301 |
| #4 | TITLE-ABS-KEY ("Medical Students" OR "Pharmacy Students" OR "Nursing students" OR "Premedical Students" ) | 118365 |
| #5 | TITLE-ABS-KEY ( "Nurses" OR "Nurse" OR "Personnel Nursing" OR "Nursing Personnel" OR "Registered Nurses" OR "Nurses Registered" OR "Registered Nurse" ) | 431676 |
| #6 | TITLE-ABS-KEY ("Pharmacist" OR "Pharmacist, Clinical" OR "Clinical Pharmacists" OR "Clinical Pharmacist" OR "Pharmacists, Clinical" OR "Hospital Pharmacist" ) | 92568 |
| #7 | #1 OR #2 OR #3 OR #4 OR #5 OR #7 | 1494578 |
| #8 | TITLE-ABS-KEY ("Mobile application" OR "Mobile applications" OR "Mobile Apps" OR "Mobile App" OR "Portable Electronic Apps" OR "Portable Software Apps" ) | 46769 |
| #9 | #7 AND #8 | 2561 |

1. Cochrane Search Strategy

| **Search** | **Query** | **Items found** |
| --- | --- | --- |
| #1 | MeSH descriptor: [Mobile Applications] explode all trees | 671 |
| #2 | MeSH descriptor: [Health Personnel] explode all trees | 8923 |
| #3 | MeSH descriptor: [Pharmacists] explode all trees | 585 |
| #4 | MeSH descriptor: [Nursing] explode all trees | 3283 |
| #5 | MeSH descriptor: [Physicians] explode all trees | 2044 |
| #6 | MeSH descriptor: [Allied Health Personnel] explode all trees | 1183 |
| #7 | #2 OR #3 OR #4 OR #5 OR #6 | 11563 |
| #8 | #1 AND #7 | 38 |
