# Supplementary material for: Mobile applications in medical education: A systematic review and meta-analysis
Source: PLoS One. 2022 Mar 24;17(3):e0265927. doi: 10.1371/journal.pone.0265927 (PMC8947018; doi:10.1371/journal.pone.0265927)
Supplement: S2 Appendix — (DOCX) [file pone.0265927.s002.docx]

**S2 Appendix: Characteristics of included studies**

**Characteristics of studies discussed on Knowledge domain**

| **Study number** | **Reference, year & country** | **Study design &population** | **Number of participants (I: Intervention; C: Control)** | **Apps used/ Characteristics of intervention and Comparison** | **Outcome measure/ Scale/ Criteria used** | **Reported limitations** |
| --- | --- | --- | --- | --- | --- | --- |
| 1 | Bonabi et al, 2019, Iran ^14^ | RCT; PHS Physicians | 107; I: 57; C:50  Final evaluation: 86; I: 43; C:43 | **Int:** Mobile app (pediatric oral health care)  **Cont:** Traditional CME (Booklet, Q&A, Pamphlet) | self-administered questionnaire (Pre & post intervention) | Less sample size; Long term effectiveness is not measured; self-administered questionnaire may cause social desirability bias |
| 2 | Velasco et al, 2015, Brazil ^15^ | RCT; Residents, Nurses &Emergency physicians | 66; I: 33; C: 33 | **Int**: Mobile app (Steps of inhalation therapy)  **Cont**: Written material | Pre- post test | Not reported |
| 3 | Clavier et al, 2019, France ^16^ | RCT; Anesthesiology residents (I & II years) | 62 (I: 32;  C: 30);  Final evaluation:  44 (I:22; C:22) | **Int**: WhatsApp (Delivery of teaching documents; Discussion of clinical cases with supervisor)  **Cont**: Traditional e learning computer platform (Access to clinical cases without live interaction with teacher) | Pre-post test (24 SCTs and 30 MCQs) | Low Cronbach alpha in SCT; Significant dropout rate; Cross-communication among students; Satisfaction questionnaire was not pre-validated |
| 4 | Noll et al, 2017, Germany ^17^ | RCT ; Medical students (UG: III year) | 44;  Group A:22; Group B: 22 | **Group A:** Mobile app (mARble-Derma [dermatology];  **Group B**: Mobile app (mble-Derma [dermatology]) | Pre- post test (10 Single Choice questions) | sample size with a power of 0.8; heterogeneous random allocation of female and male participants to the two groups |
| 5 | Samra et al, 2016, USA ^18^ | RCT; Medical students (UG: III year) | 29; I: 15; C: 14  Final evaluation 21; I: 7, C:14 | **Int**: Regular class + Buckingham Virtual Tympanum iPhone app (Tympanic Membrane anatomy & pathology)  **Cont**: Regular class | Pre-post test (Max. score: 16) | Not reported |
| 6 | Albrecht V et al, 2013, Germany ^19^ | RCT; Medical students (UG: III year) | 10; I: 6  C: 4 | **Int**: mARble Forensics (Forensic module)  **Cont**: Textbook material | Pre- post test (10 Single questions) | Less sample size; Social interaction or missing interaction during the learning process |
| 7 | Stirling et al, 2014, Australia ^20^ | RCT; Medical students (UG: III year) | 71; C: 33  I: 38 | **Int**: eBook App (Gross anatomy of the human heart)  **Cont**: Traditional practical session | Pre-post test (15 MCQs) | Less sample size; Less study duration; limitation on the depth and quantity of questions used |
| 8 | Amer et al, 2017, USA ^21^ | RCT; Medical students (UG: II & III year) | 100; C: 50  I: 50 | **Int**: Touch-Surgery application (CTR surgery)  **Cont**: Video lecturing using slides | Post test (21 MCQs) | Actual surgical dexterity or technique was not tested; choice of the control group’s teaching medium (Audio dubbed slides); restriction of generalizability only to the medical students. |
| 9 | Kucuk et al, 2016, Turkey ^22^ | RCT; Medical students (UG: II year) | 70; I: 34  C: 36 | **Int**: mAR application (Neuroanatomy)  **Cont**: Traditional teaching (using 2D pictures, graphs and text) | Academic achievement  test (30 MCQs) | The necessity of internet connection for mAR applications; Incompatibility of mAR software with some smart phones |
| 10 | Brown et al, 2018, USA ^23^ | RCT; Medical students (UG: II year) | 67 | **Int**: LK app (Pathology of Vasculitides and Atherosclerosis)  **Cont**: Written notes | Pre -post test (10 MCQs) | Because of software compatibility issues, only iPhone users could be accepted in the intervention group. |
| 11 | Lacy et al, 2018, USA ^24^ | RCT; Medical students (UG) | 36 | **Int**: Skinder app (Benign and malignant skin lesions images)-  **Cont**: ABCD rule-based algorithm (traditional practice) | Pre-post test (32 image test) | Not reported |
| 12 | Fernandes Pereira et al, 2016, Brazil ^25^ | RCT; Nursing students (UG: II semester) | 100; C: 50  I: 50 | **Int**:CalcMed application (For medicament Calculation)  **Cont**: Conventional method (Maths skills and calculator) | Post-test (10 questions) | Low quality evaluation criteria for application as well as student’s performance |
| 13 | Putri et al, 2019, Indonesia ^26^ | RCT; Nursing students (UG) | 48; I: 24; C: 24 | **Int:** Life Saving: Learning and Guideline application (BLS Concepts)  **Cont:** Traditional lectures | Pre-post test (20 questions) | Not reported |
| 14 | Wang et al, 2017,  China ^27^ | Cluster RCT; Registered nurses in primary care setting | 115; I: 61  C: 54 | We chat (For online  Interaction with peers)  **Int**: Lecture + face to face discussion + we chat  **Con**: Lecture + face to face discussion | CADKS (30 true/false questions) | Limited to a region in China; Self-reported questionnaire; Identifying the component of the intervention is really effective due to the use of combination of face-to-face and WeChat educational intervention |
| 15 | Ziabari et al, 2019, Iran ^28^ | Quasi-experimental study; Medical students (UG: Interns) | 100; I: 50;  C: 50 | **Int**: Regular education + Telegram app (Continuing education on BLS)  **Cont**: Regular education | Pre -post test (BLS awareness score- 20 questions) | Lack of randomization and the impossibility of blinding; Lack of facilities for examining the practical skills of students at the same time |
| 16 | Briz-Ponce et al, 2016,  Spain ^29^ | Quasi-experimental study; Medical students (UG) | 30;I: 15; C: 15 | **Int**: Anatomic app (Human brain)  **Cont**: Traditional class | Pre-post test | Not reported |
| 17 | Golshah et al, 2020, Iran ^30^ | Quasi-experimental study; Dental students (UG) | 53; I: 27; C: 26 | **Int:** Mobile app (Radiographic Cephalometry)  **Cont:** Traditional lecture-based instruction | Post test (Cephalometric landmark identification) | Small sample size |
| 18 | Salameh et al, 2020, Palestine ^31^ | Quasi-experimental study; Nursing students (Critical care) | 104; I: 52; C: 52 | **Int:** Mobile app (ECG analysis, arrhythmia interpretations and management  **Cont:** Traditional learning material | Pre-post quiz (20 MCQs) | Results cannot be generalized, because of findings from one topic |
| 19 | Chung et al, 2018, USA ^32^ | Interventional cohort study; Medical residents (III year Peadiatrics) | 37; I: 18  C: 19 | **Int:** Audience response system (ARS) app (Bedside education in PICU)  **Cont**: Traditional bedside teaching | Pre -post test (50 free text questions) | Incompletion of all 10 modules due to rotaion in their rounds,; limited number of questions; Non-randomized study design |
| 20 | Deshpande et al, 2017, India ^33^ | Interventional cohort study; Dental students (Interns) | 92 (Single group) | **Int:** Mobile app (Prosthodontics – 35 real patient cases) | SCT (60 questions) | Small sample size; Single centre study |
| 21 | Man et al, 2014, USA ^34^ | Interventional cohort study; Physicians | N= 14 (Single group) | **Int**: Smart phone app ( For guiding Antidepressant Drug Selection) | Pre post survey (15 items) | Application is limited to major depressive disorder only. |
| 22 | Liu et al, 2018, Taiwan ^35^ | Interventional cohort study; Medical students (Dermatology residents, non-dermatology trainees) | 26;  Group 1:13;  Group 2: 13 | **Int**: Wallpaper-changing software application (Identification of medical fungi)  **Group 1**: Dermatology residents.  **Group 2**: Non dermatology trainees | Pre- post test  (Pre test: 20 MCQs; Post test: 40 MCQs) | Les number of dermatology residents; intensity of exposure was non-quantifiable; Android limitation operating systems |
| 23 | Fralick et al, 2017, Canada ^36^ | Interventional cohort study; Medical students (Senior medical students & residents) | 62; I: 32; C: 30;  Follow up: 53; I: 27; C: 26 | **Int**: Smartphone app (hospital’s antibiogram and treatment guidelines)  **Cont**: No application | Pre-post test  (12 MCQs/ true or false questions) | Non-validated knowledge assessment test; Single center study; short duration of follow-up; non- randomized design and unmeasured confounding factors |
| 24 | Weldon et al, 2019, UK ^37^ | Interventional cohort study; Medical/ Veterinary/ Life science PG students | 5 (Single group) | **Int**: 3D visualisations app (Obsessive compulsive disorder) | Pre- post test (6 MCQs) | Limited period of time; Small number of participants;  Short and simple survey |
| 25 | Smeds et al, 2016, USA ^38^ | Interventional cohort study; Medical students (UG: III year) | 288; I: 152 ; C: 136 | **Int**: Spaced education mobile app (General surgery based MCQs)  **Cont**: No application | NBME Score, USMLE Score, GPA, MCAT Score | Single-center design;  Usage of application for a brief time |
| 26 | Hirunyanitiwattana et al, 2020, Thailand ^39^ | Interventional cohort study; Nurses | 44; I: 25; C: 19 | **Int:** Asthma Care Application (Asthma education)  **Cont:** Written Asthma Action Plan | Pre-post test (General Asthma Knowledge: 15 items; Asthma Action Plan Knowledge: 15 items) | Single centre study; Small sample size; High pre test scores of the participants; |
| 27 | Baccin et al, 2020, Brazil ^40^ | Interventional cohort study; Nurses & nursing students (UG) | 161  Final evaluation: 150 | **Int:** mSmartAVC (Nursing care for ischemic and hemorrhagic CVA) | Pre-post test (20 MCQs) | Difficulty to access Wi-Fi for downloading app in university; Resistance from administrators for using the app near bedside; Human & environmental barriers |
| 28 | Ameri et al, 2020, Iran ^41^ | Interventional cohort study; Pharmacy students (UG) | 316; C: 106  Group 1: 105  Group 2: 105 | **Int:** LabSafety application (Safety measures in pharmaceutical labs)  **Cont:** No education  **Group 1:** Traditional education using paper handouts & instructions  **Group 2:** Education using LabSafety application | Pre-post test (20 MCQs) | Android compatible app developed, didn’t check compatibility with other software platforms; Long term effects is not studied; Single centre study |
| 29 | Hisam et al, 2019, Pakistan ^8^ | Cross-sectional study; Medical students (UG: II,III,IV,V years); | 448  I: 323 ; C: 125 ; | **Int**:Using any medical application (Medscape; Visual Anatomy; Pharmapedia; Oxfords Medical Dictionary; Medical Pneumonic; Disease Dictionary)  **Cont**: Not using medical application | Score in the last professional examination | Convenient nonprobability  sampling technique and short span of time |

2D: 2 dimensional; 3D: 3 dimensional; BLS: Basic life support; CADKS: Chinese Alzheimer's Disease Knowledge Scale; CME: Continuing Medical Education; CTR: Carpal Tunnel Release; GPA: Grade point average; LK: Lecture Keepr; mAR: Mobile augmented reality; mARble: mobile Augmented Reality blended learning environment; mble: mobile blended learning environment; MCAT: Medical College Admissions Test; MCQ: multiple-choice question; NBME: National Board of Medical Examiners; PHS: Public Health Service; PICU: Pediatric Intensive Care Unit; PG: Post Graduate; RCT: Randomized controlled trial; SCT: Script Concordance Test; UK: United Kingdom; UG: Undergraduate; USA: United States of America; USMLE: United States Medical Licensing Examination

**Characteristics of studies discussed on Skill domain**

| **Study number** | **Reference, year & country** | **Study design & population** | **Number of participants** | **Apps used/ Characteristics of intervention and Comparison** | | **Outcome measure/Scale/Criteria used** | **Reported limitations** |
| --- | --- | --- | --- | --- | --- | --- | --- |
| 1 | Nadir et al, 2019, USA ^42^ | RCT; Medical students (Post graduate residents) | 58; C:29  I: 29 | **Int**: airRx (simulated  in-flight medical emergencies)  **Cont**: No application | | Check list, GRS  (2 cases: Syncope & Shortness of breath) | Small amount of time learners interacted with the app before using it; Did not control for the confounding variable of other app usage; Simulation scenarios were short at 8 minutes; Non-blinded raters |
| 2 | Mamtora et al, 2018, UK ^43^ | RCT; Medical students (UG: Final year) | 20; C: 10  I: 10 | **Int**: D-EYE (ophthalmoscope attached to mobile application)  **Cont**: Direct ophthalmoscope | | Objective questionnaire (60 points) | Non-blinded nature; Students examined the same film slides twice using each imaging modality, which could influence the findings when examining the same slide for the second time. |
| 3 | Haubruck et al, 2018, UK ^44^ | RCT; Medical students (UG: III-VI years) | 95; I: 49  C: 46 | **Int**: Touch Surgery (Teaching chest tube insertion)  **Cont**: Thoracocentesis training | | OSATS | Subjective self-evaluation (evaluation of motivation and satisfaction) |
| 4 | Oliveira et al, 2019, Brazil ^45^ | RCT; Dental students (UG: IV, V years) | 20; C: 10  I: 10 | **Int**: Kahoot (Radiographic images of endodontic conditions),  **Cont**: No application | | Diagnostic ability (10 Conditions) | Single centre study |
| 5 | Strandell-Laine et al, 2018, Finland ^46^ | RCT; Medical students (Beginners of five week internal medicine or surgical clinical practicum; 2^nd^ pre-registration nursing) | 102; I= 52  C = 50 | **Int**: Study@CampusPro (For enhancing competence and self efficacy)  **Cont**: No application | | NCS, SECP | Non-blinding among the participants; Single district based center implementation of intervention |
| 6 | Bartlett et al, 2017, UK ^47^ | RCT; Medical students (UG: IV & V years) | 27; Group 1: 9, Group 2: 9, Group 3: 9 | **Int:** Touch Surgery (Male urinary catheterization scenario)  **Group 1**: No formal revision resources  **Group 2**: Traditional revision resources  **Group 3**: Touch Surgery | OSCE (46 points) | | Single centre study; Students were only recruited from years 4^th^& 5^th^ years; Single scenario based study; Limited sample size; |
| 7 | Low et al, 2011, UK ^48^ | RCT; Junior doctors (Resuscitation Council ALS-trained doctors (within 5 years of qualification) | 31; I: 16; C: 15 | **Int:** iResus (Advanced life support: Bradycardia algorithm)  Cont: No application | | CASTest (Max score: 96) | Both groups received training on the ‘app’, but control did not had access to the ‘app’ during their assessment scenario; Assessors were not blinded; Limited training period |
| 8 | Miriam McMullan, 2018, UK ^49^ | Interventional cohort study; Paramedics &ODP students (UG: II year) | 60; Paramedics: 41; ODP: 19 | **Int**: Medication calculation mobile app | | Pre -post test | Lack of a control group; Apps instructional design and learners of different levels of expertiseis not evaluated |
| 9 | Meyer et al, 2018, USA ^50^ | Interventional cohort study; Internal medicine physicians | 46 | **Int**: PTT advisor application;  **Cont**: Usual clinical decision support | | Percent of test ordering and correct diagnosis (368vignettes) | Participant bias due to their high level of experience and comfort; Not assessed the effectiveness using real cases |
| 10 | Quezada et al, 2019, Chile ^51^ | Interventional cohort study; Surgical trainees | 55; C: 25  I: 30 | **Int**: Lapp (Laproscopic skill course)  **Cont**: Traditional in person simulation training | | GRS, SRS | Non-randomized study design |

CASTest: Cardiac Arrest Simulation Tests; GRS: Global rating scale; NCS: Nurse Competence Scale; ODP: Operating Department Practice; OSATS: Objective Structured Assessment of Technical Skills; PTT: Partial thromboplastin times; RCT: Randomized controlled trial; SECP: Self-Efficacy in Clinical Performance; SRS: Specific rating scale; UG: Undergraduate; UK: United Kingdom; USA: United States of America

**Characteristics of studies discussed on both Knowledge & Skill domain**

| **Study number** | **Reference, year & country** | **Study design & population** | **Number of participants** | **Apps used/ Characteristics of intervention and Comparison** | **Outcome measure/Scale/Criteria used** | **Reported limitations** |
| --- | --- | --- | --- | --- | --- | --- |
| 1 | Martínez et al, 2017, Chile ^9^ | RCT; Medical students (UG: Interns) | 80; I: 40; C: 40 | **Int:** Mobile application based additional training for EUNACOM  **Cont:** No additional training for EUNACOM | Final test score of EUNACOM | Significant proportion of students did not attend the final examination (18.7%); Impossibility to mask participants to the intervention; sharing of contents across the study groups |
| 2 | Naveed et al, 2018, UK ^52^ | RCT; Medical students (UG) | 20; C: 10, I: 10; Final evaluation (C: 7, I: 8) | **Int**: BaSSiS mobile simulation App (Basic concepts and techniques of skin surgery)  **Cont**: Traditional text-based self-study | MCQs (33 questions),  OSATS | Assurance on sincerity of participants in the study; small sample size; small difference in average scores |
| 3 | Kim et al, 2018, South Korea ^53^ | RCT; Nursing students (Senior students) | 72; C: 36  I: 36  Final evaluation (C: 32, I: 34) | **Int**: Interactive Clinical Nursing Skills Mobile Application  **Cont**: Non-interactive Clinical Nursing Skills Mobile Application | Pre - post test | Single cohort; Lack of generalizability of findings; Did not completely eliminate the effects of diffusion between the groups; Did not include full range of clinical nursing skills; long-term effects are not measured; Lack of internal consistency of knowledge scale |
| 4 | Bayram et al, 2019, Turkey ^54^ | RCT; Fundamentals of Nursing II course students (UG: First year) | 118; C: 59  I:59 | **Int**: Tracheostomy care regular class + Game based virtual reality phone Application (tracheostomy care)  **Cont**: Tracheostomy care regular class | Pre post test | Intervention period: 7 days only |
| 5 | Fernández-Lao et al, 2016, Spain ^55^ | RCT; Physiotherapy students | 49; I: 25  C: 24 | **Int**: Regular theory & practical class + Mobile app (Palpation and ultrasound skills in the shoulder area).  **Cont**: Regular theory & practical class | MCQs (20 questions),  OSCE | Single center experience; Less generalizability of findings to other subjects and languages because of single centre study |
| 6 | Lozano-Lozano et al, 2020, Spain ^56^ | RCT: Physiotherapy students (UG) | 110; C: 55, I: 55  Final Evaluation: C: 55, I: 50 | **Int:** Ecofisio mobile app (sports pathology)  Cont: Traditional learning | MCQ, OSCE | Results could not be generalized as it enrolled students from 2 universities only; No follow up to evaluate the sustainability |
| 7 | Bunogerane et al, 2017, Rwanda ^57^ | RCT; Surgery residents (General surgery, Orthopedics, Urology & Neurosurgery | 27;  C: 13; I: 14 | **Int**: Touch Surgery application (Tendon repair)  **Cont**: Text book | Pre - post test (MCQs), Simulation test score | Small sample size; Lack of formal calculation of inter-rater reliability; |
| 8 | Kang et al, 2018, South Korea ^58^ | Quasi-experimental  study; Nursing students (UG: III year) | 92; I: 49 (HTN: 21; DM: 28)  C: 43 (HTN: 20; DM: 23) | **Int**: HTN App, DM app  **Cont**: No application | Pre-Post test (27 questions) | Limited availability (compatible with android only); Non-Randomized allocation of participants; Single Center; Limited generalizability of findings |
| 9 | Young Yoo et al, 2015, South Korea ^59^ | Quasi-experimental  study; Nursing students (UG: II year) | 22 (11 each cross over) | **Int**: Mobile app (Lungs, heart simulation exercises)  **Cont**: Human patient simulator | Pre-Post test;  Clinical assessment skill | Limited use (Single day) of the high-fidelity human patient simulator;  Inconvenience in additional practice of human patient simulator |
| 10 | Kim et al, 2017, South Korea ^60^ | Quasi-experimental  study ; Nursing students (UG: III year) | 80; I: 40; C: 40  Final evaluation (I: 35; C:38) | **Int:** Caring for infants with airway obstruction application  **Cont:** One time lecture | Pre – post test (8 MCQs; 6 true or false questions);  Checklist (22 points) | Did not check how many times students used the application; Did not use standardized measures; Small sample size; Feedback was not collected after using intervention |
| 11 | Kang et al, 2020, South Korea ^61^ | Quasi-experimental  study ; Nursing students (UG) | 86;  Exp 1: 26; Exp 2: 32; Cont: 28 | **Int:** Smart phone app (Breast self examination)  Exp 1: Smartphone app education;  Exp 2: Smartphone app education + hands-on practice;  Cont: Lecture-based education | Pre – post test (Knowledge: 22 questions; Skills 13 questions) | Single centre study; Retention of knowledge didn’t assessed; Not RCT; Convenience sampling method |
| 12 | Shore et al, 2018, USA ^62^ | Interventional cohort study; Medical residents (Orthopedic residents) | 53 (single group) | **Int**: Touch Surgery application (Septic hip arthritis) | Pre - post test (32 MCQs), Simulation test score | Small cohort; Short study duration;  Selection bias (failed to note participants past experience in study topic and motivation level) |
| 13 | [Ebner](https://www.ncbi.nlm.nih.gov/pubmed/?term=Ebner%20F%5BAuthor%5D&cauthor=true&cauthor_uid=31042155) et al, 2019, Germany ^63^ | Interventional cohort study; Medical students (UG) | 66; I: 33; C: 33 | **Int**: Textbook + Ultraschall App (Kidney ultrasound training)  **Cont**: Textbook | MCQs (range 0-6 points); Accurate kidney scan | Non-random allocation; Not differentiated between preexisting motor skills and app-trained skills |

DM: Diabetes mellitus; EUNACOM: Examen Unico Nacional de Conocimientos de Medicina (National Examination of Medical Knowledge, is a theoretical-practical examination of general medicine in Chile); HTN: Hypertension; MCQ: Multiple-choice question; OSATS: Objective Structured Assessment of Technical Skills; OSCE: Objective structured clinical evaluation; RCT: Randomized controlled trial; UG: Undergraduate
