# Supplementary figures and images for: Mobile applications in medical education: A systematic review and meta-analysis
Source: PLoS One. 2022 Mar 24;17(3):e0265927. doi: 10.1371/journal.pone.0265927 (PMC8947018; doi:10.1371/journal.pone.0265927)

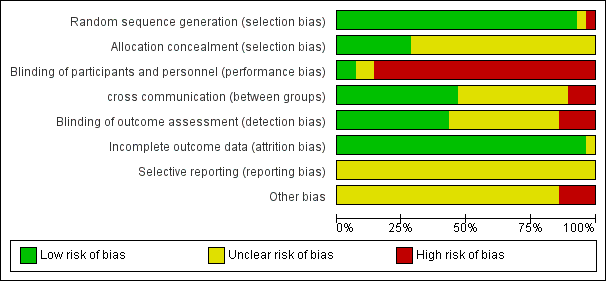

Supplement: S3 Appendix — (PNG) [file pone.0265927.s003.png]

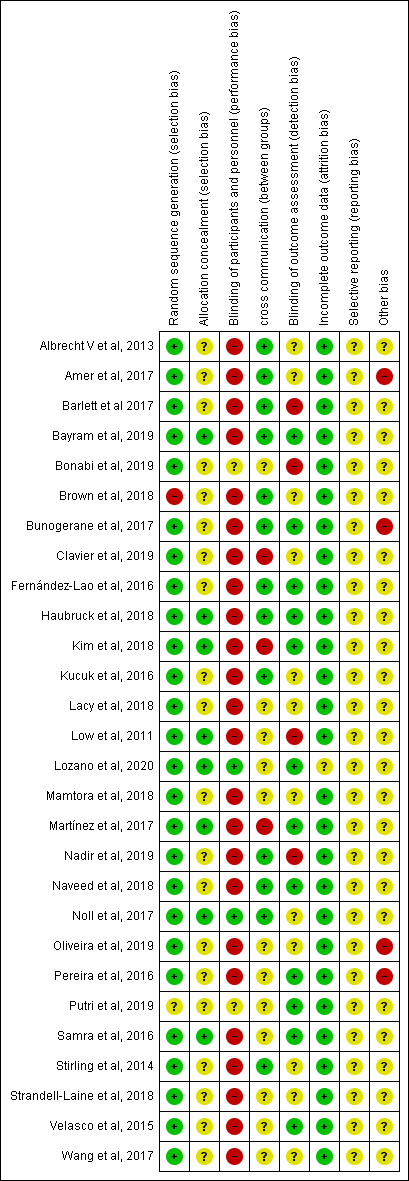

Supplement: S4 Appendix — (PNG) [file pone.0265927.s004.png]

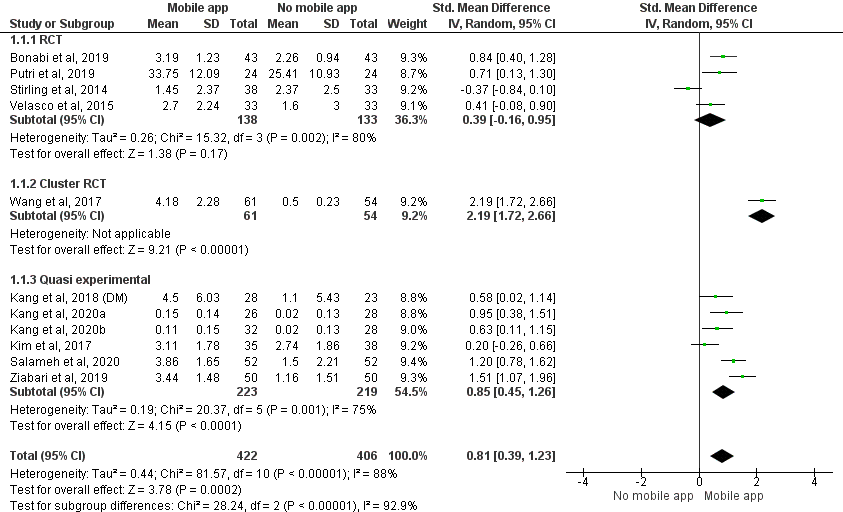

Supplement: S6 Appendix — (PNG) [file pone.0265927.s006.png]

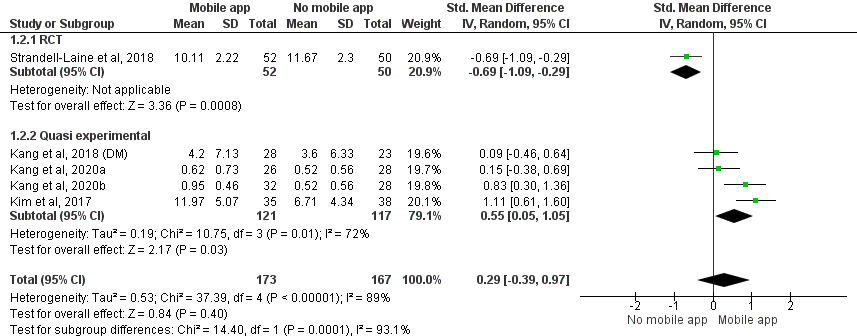

Supplement: S7 Appendix — (PNG) [file pone.0265927.s007.png]

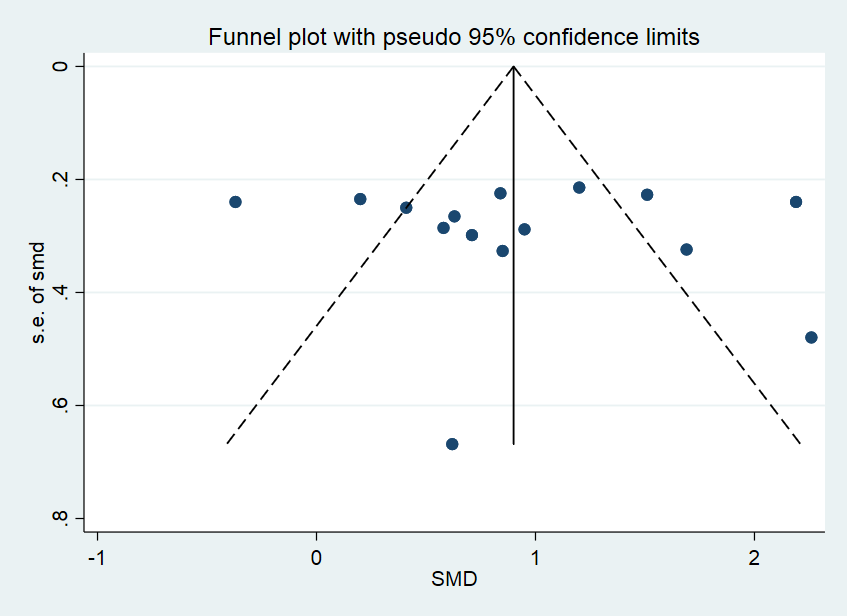

Supplement: S8 Appendix — (PNG) [file pone.0265927.s008.png]

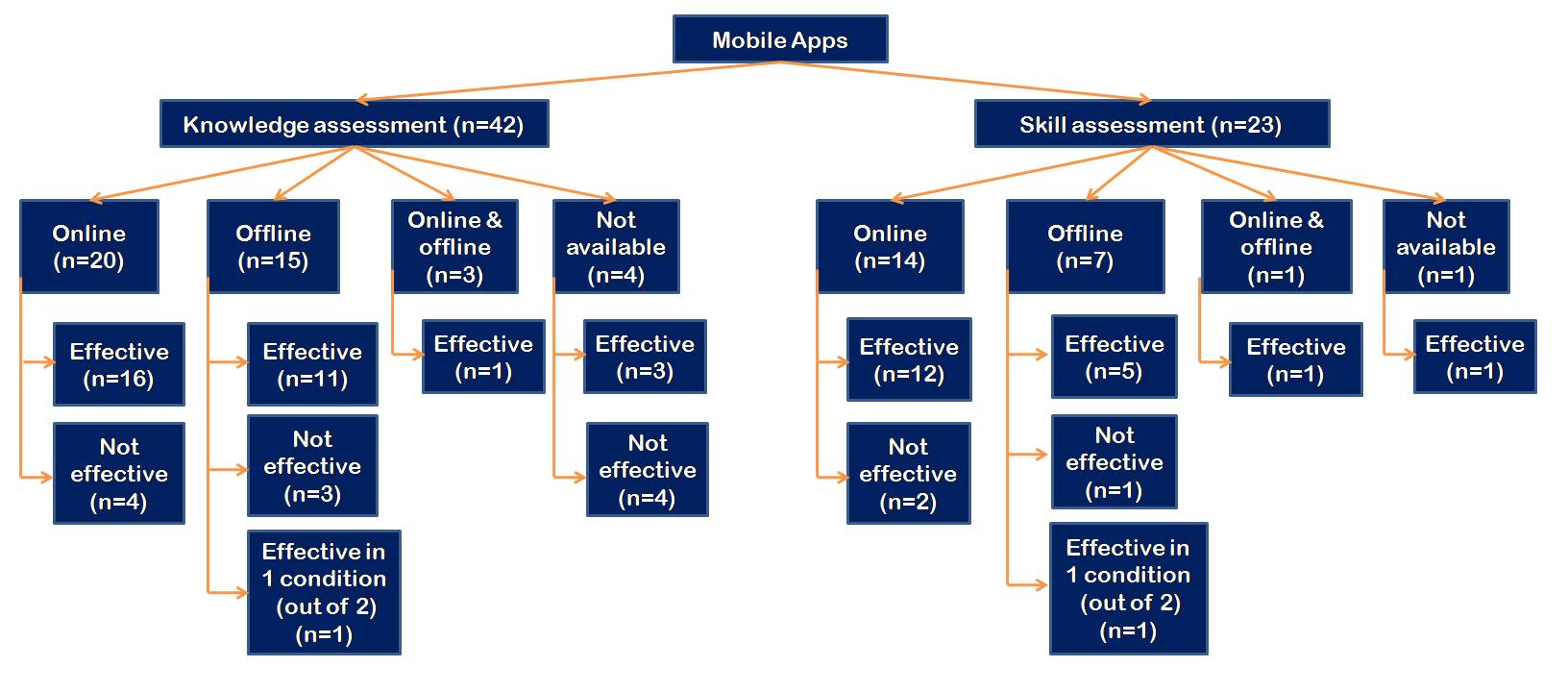

Supplement: S9 Appendix — (JPG) [file pone.0265927.s009.jpg]
