# Supplementary material for: Mobile applications in medical education: A systematic review and meta-analysis
Source: PLoS One. 2022 Mar 24;17(3):e0265927. doi: 10.1371/journal.pone.0265927 (PMC8947018; doi:10.1371/journal.pone.0265927)
Supplement: S5 Appendix — (DOCX) [file pone.0265927.s005.docx]

**S5 Appendix: New castle Ottawa** **Score of included studies**

| **Author, year & country** | **Representativeness of intervention group** | **Selection of comparison group** | **Comparability of comparison group** | | **Study retention** | **Blinding** | **Total score** |
| --- | --- | --- | --- | --- | --- | --- | --- |
|  |  |  | **Non Randomized study** | | *Lost to follow-up loss  (< 25%)* | *Was Outcome assessment blinded?* |  |
|  |  |  | *Baseline scores* | *Baseline characteristics* |  |  |  |
| Ziabari et al, 2019, Iran | 1 | 1 | 1 | 1 | 1 | NA | **5** |
| Briz-Ponce et al, 2016,  Spain | 1 | 1 | 1 | 1 | 1 | NA | **5** |
| Chung et al, 2018, USA | 1 | 1 | 1 | 0 | 1 | 0 | **4** |
| Deshpande et al, 2017, India* | 1 | 0 | 0 | 0 | 1 | 1 | **3** |
| Man et al, 2014, USA* | 1 | 0 | 0 | 0 | 1 | 0 | **2** |
| Liu et al, 2018, Taiwan | 1 | 1 | 0 | 0 | 1 | 1 | **4** |
| Fralick et al, 2017, Canada, | 1 | 1 | 1 | 1 | 1 | 1 | **6** |
| Weldon et al, 2019, UK* | 1 | 0 | 0 | 0 | 1 | 1 | **3** |
| Smeds et al, 2016, USA | 1 | 1 | 0 | 0 | 1 | 1 | **4** |
| Hisam et al, 2019, Pakistan | 1 | 1 | 0 | 0 | 1 | 1 | **4** |
| Golshah et al, 2020, Iran | 1 | 1 | 1 | 1 | 1 | 0 | **5** |
| Salameh et al, 2020, Palestine | 1 | 1 | 0 | 1 | 1 | 1 | **5** |
| Kang et al, 2020, South Korea | 1 | 1 | 1 | 1 | 1 | 1 | **6** |
| Hirunyanitiwattana et al, 2020, Thailand | 1 | 1 | 1 | 1 | 1 | 1 | **6** |
| Baccin et al, 2020, Brazil* | 1 | 0 | 0 | 0 | 1 | 1 | **3** |
| Ameri et al, 2020, Iran | 1 | 1 | 1 | 1 | 1 | 1 | **6** |
| Miriam McMullan, 2018, UK* | 1 | 0 | 0 | 0 | 1 | 1 | **3** |
| Meyer et al, 2018, USA | 1 | 1 | 0 | 0 | 1 | 1 | **4** |
| Quezada et al, 2019, Chile | 1 | 1 | 1 | 1 | 1 | 1 | **6** |
| Kang et al, 2018, South Korea | 1 | 0 | 1 | 1 | 1 | 0 | **4** |
| Young Yoo et al, 2015, South Korea | 1 | 1 | 1 | 1 | 1 | NA | **5** |
| Kim et al, 2017, South Korea | 1 | 1 | 1 | 1 | 1 | 1 | **6** |
| Shore et al, 2018, USA* | 1 | 0 | 0 | 0 | 1 | 1 | **3** |
| [Ebner](https://www.ncbi.nlm.nih.gov/pubmed/?term=Ebner%20F%5BAuthor%5D&cauthor=true&cauthor_uid=31042155) et al, 2019, Germany | 1 | 1 | 1 | 1 | 1 | 1 | **6** |

USA: United States of America; NA: Not available; UK: United Kingdom

*Represent single group studies
