# Supplementary material for: Mobile applications in medical education: A systematic review and meta-analysis
Source: PLoS One. 2022 Mar 24;17(3):e0265927. doi: 10.1371/journal.pone.0265927 (PMC8947018; doi:10.1371/journal.pone.0265927)
Supplement: S10 Appendix — (DOCX) [file pone.0265927.s010.docx]

**S10 Appendix: Mobile application operating system**

| **Mobile application operating system** | | | | | | | | | |
| --- | --- | --- | --- | --- | --- | --- | --- | --- | --- |
| **Knowledge assessment (n=42)** | | | | | **Skill assessment (n=23)** | | | | |
| Particulars | Android (n=10) | iOS (n=9) | Both Android and iOS (n=16) | Not mentioned (n=7) | Particulars | Android (n=3) | iOS (n=5) | Both Android and iOS (n=11) | Not mentioned (n=4) |
| Effective | 8 | 7 | 14 | 4 | Effective | 2 | 5 | 10 | 2 |
| Not effective | 2 | 2 | 2 | 2 | Not effective | 1 | 0 | 0 | 2 |
| Effective in one condition (out of 2) | 0 | 0 | 0 | 1 | Effective in one condition (out of 2) | 0 | 0 | 1 | 0 |
